# The long non-coding RNA nuclear-enriched abundant transcript 1_2 induces paraspeckle formation in the motor neuron during the early phase of amyotrophic lateral sclerosis

**DOI:** 10.1186/1756-6606-6-31

**Published:** 2013-07-08

**Authors:** Yoshinori Nishimoto, Shinichi Nakagawa, Tetsuro Hirose, Hirotaka James Okano, Masaki Takao, Shinsuke Shibata, Satoshi Suyama, Ken-ichiro Kuwako, Takao Imai, Shigeo Murayama, Norihiro Suzuki, Hideyuki Okano

**Affiliations:** 1 Department of Physiology, School of Medicine, Keio University, 35 Shinanomachi, Shinjuku-ku, Tokyo 160-8582, Japan; 2 Department of Neurology, School of Medicine, Keio University, 35 Shinanomachi, Shinjuku-ku, Tokyo 160-8582, Japan; 3 RNA Biology Laboratory, RIKEN Advanced Science Institute, 2-1 Hirosawa, Saitama 351-0198, Japan; 4 Genomic Neo-Function Research Group, Biomedical Research Institute, National Institute of Advanced Industrial Science and Technology (AIST), 2-4-7 Aomi, Koutou-ku, Tokyo 135-0064, Japan; 5 Division of Regenerative Medicine, Jikei University School of Medicine, 3-25-8 Nishi-Shinbashi, Minato-ku, Tokyo 105-8461, Japan; 6 Institute of Brain and Blood Vessels, Mihara Memorial Hospital, 366 Ohtacho, Isesaki, Gunma 372-0006, Japan; 7 Tokyo Metropolitan Geriatric Hospital & Institute of Gerontology, 35-2 Sakaecho, Itabashi, Tokyo 173-0015, Japan

**Keywords:** Long non-coding RNA, Paraspeckle, NEAT1_2, TDP-43, FUS/TLS, Amyotrophic lateral sclerosis, Electron microscopy

## Abstract

**Background:**

A long non-coding RNA (lncRNA), nuclear-enriched abundant transcript 1_2 (NEAT1_2), constitutes nuclear bodies known as “paraspeckles”. Mutations of RNA binding proteins, including TAR DNA-binding protein-43 (TDP-43) and fused in sarcoma/translocated in liposarcoma (FUS/TLS), have been described in amyotrophic lateral sclerosis (ALS). ALS is a devastating motor neuron disease, which progresses rapidly to a total loss of upper and lower motor neurons, with consciousness sustained. The aim of this study was to clarify the interaction of paraspeckles with ALS-associated RNA-binding proteins, and to identify increased occurrence of paraspeckles in the nucleus of ALS spinal motor neurons.

**Results:**

*In situ* hybridization (ISH) and ultraviolet cross-linking and immunoprecipitation were carried out to investigate interactions of NEAT1_2 lncRNA with ALS-associated RNA-binding proteins, and to test if paraspeckles form in ALS spinal motor neurons. As the results, TDP-43 and FUS/TLS were enriched in paraspeckles and bound to NEAT1_2 lncRNA directly. The paraspeckles were localized apart from the Cajal bodies, which were also known to be related to RNA metabolism. Analyses of 633 human spinal motor neurons in six ALS cases showed NEAT1_2 lncRNA was upregulated during the early stage of ALS pathogenesis. In addition, localization of NEAT1_2 lncRNA was identified in detail by electron microscopic analysis combined with ISH for NEAT1_2 lncRNA. The observation indicating specific assembly of NEAT1_2 lncRNA around the interchromatin granule-associated zone in the nucleus of ALS spinal motor neurons verified characteristic paraspeckle formation.

**Conclusions:**

NEAT1_2 lncRNA may act as a scaffold of RNAs and RNA binding proteins in the nuclei of ALS motor neurons, thereby modulating the functions of ALS-associated RNA-binding proteins during the early phase of ALS. These findings provide the first evidence of a direct association between paraspeckle formation and a neurodegenerative disease, and may shed light on the development of novel therapeutic targets for the treatment of ALS.

## Background

Amyotrophic lateral sclerosis (ALS) is a devastating neurodegenerative disorder characterized by loss of upper and lower motor neurons. The clinical symptoms of ALS typically develop at between 50 to 70 years of age, leading to skeletal muscle weakness including respiratory failure. The overall median tracheostomy free survival is 2.5 years [1]. Among the genes associated with familial ALS, mutations in TAR DNA-binding protein-43 (TDP-43), fused in sarcoma/translocated in liposarcoma (FUS/TLS), optineurin and SQSTM1, and hexanucleotide repeat expansion in C9ORF72 were also identified in sporadic ALS cases [2-7]. Wild-type (WT) TDP-43 and FUS/TLS are predominantly observed in the nucleus by immunostaining [8,9]. Besides full-length WT TDP-43, 35-kDa and 18–26-kDa C-terminal fragments are produced via caspase-dependent and -independent pathways [8,10]. The 26-kDa C-terminal TDP-43 fragment aggregated insolubly in the cytoplasm of ALS motor neurons with ubiquitination and phosphorylation [11,12]. We previously demonstrated that the 35-kDa C-terminal fragment functions in the formation of stress granules in the cytoplasm, which induces mRNA stabilization and translational arrest against stresses [8]. Similarly, FUS/TLS mutants linked with ALS, which lacked the nuclear import activity, demonstrated mislocalization to the cytoplasm and formed a stress granule-like structure [9]. TDP-43 and FUS/TLS play critical roles in RNA processing [13]; however, the association of these RNA-binding proteins with ALS pathogenesis remains mostly unknown.

As another specific finding to sporadic ALS, the A-to-I RNA editing efficiency of mRNA encoding the GluA2 subunit of the α-amino-3-hydroxy-5-methyl-4-isoxazolepropionic acid (AMPA) receptor varied greatly, from 0% to 100% among the motor neurons of sporadic ALS cases. This observation was in marked contrast to control motor neurons, all of which demonstrated 100% editing efficiency [14].

Paraspeckle is known as one of factors which have influence on edited RNAs [15,16]. Among nuclear bodies which are important for RNA processing, paraspeckle is in close proximity to nuclear speckles [17-20]. Based on bioinformatics analyses, the nuclear-enriched abundant transcript1 (NEAT1) locus generates two types of non-coding RNAs (ncRNAs) from the same promoter in the human genome: 3.7 kb NEAT1_1 (MENϵ) and 23 kb NEAT1_2 (MENβ) [21,22]. Notably, NEAT1_2 long non-coding RNA (lncRNA) is essential for paraspeckle formation [17-20]. Recent reports, using individual nucleotide-resolution ultraviolet (UV) cross-linking and immunoprecipitation (iCLIP), CLIP-seq and photoactivatable ribonucleoside-enhanced cross-linking and immunoprecipitation (PAR-CLIP) procedures, displayed that NEAT1_2 lncRNA was one of RNAs bound by both TDP-43 and FUS/TLS [23-27].

Previous electron microscopic observations indicated that the paraspeckle corresponds to a specific structure of the interchromatin granule-associated zones (IGAZ) [17,28-30]. In the current model, paraspeckles consist of NEAT1 ncRNA and more than 40 paraspeckle proteins including paraspeckle protein-1 (PSP1)/paraspeckle component1, p54^nrb^/non-POU domain-containing octamer-binding protein (NONO), polypyrimidine tract binding protein-associated splicing factor (PSF), RNA polymerase II and other proteins [16,31-34]. Among these proteins, p54^nrb^ and PSF are core paraspeckle proteins that trigger the formation of paraspeckles through an interaction with NEAT1_2 lncRNA.

Building upon these previous findings, we investigated the association of paraspeckles with TDP-43 and FUS/TLS in the nucleus and the alteration in paraspeckle formation in spinal motor neurons of ALS patients.

## Results

### TDP-43 and FUS/TLS are enriched in nuclear paraspeckles in cultured cells

**Figure 1:**
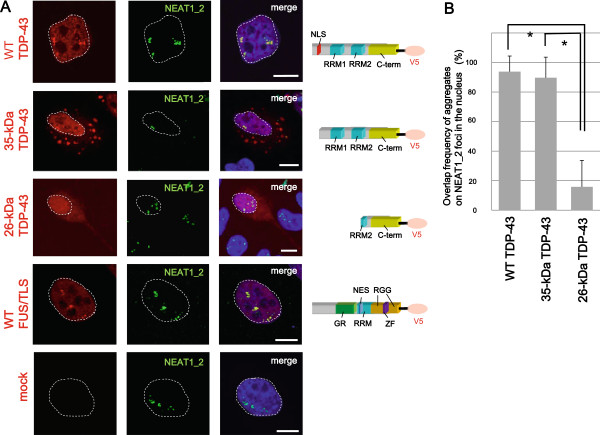
**TDP-43 and FUS/TLS aggregate at nuclear NEAT1_2 foci. A**. At 48 hours after transfection with wild-type (WT) and 35-kDa TDP-43 fragment and WT FUS/TLS with the V5 tag at the C terminus, HeLa cells were fixed with 4% paraformaldehyde, hybridized with fluorescein isothiocyanate (FITC)-labeled RNA probe against NEAT1_2 long non-coding RNA (lncRNA), and double-immunostained with polyclonal anti-FITC and monoclonal anti-V5 antibodies. Schematic diagram (right) represents the TDP-43 isoforms and FUS/TLS. NLS, Nuclear localization signal; RRM, RNA recognition motif; C-term, C-terminal domain; GR, glycine-rich motif; NES, nuclear export signal; RGG, arginine-glycine-glycine motif ; ZF, zinc-finger motif. Dotted lines represent the outline of the nucleus. Scale bars, 10μm. **B**. The Y-axis shows what percent NEAT1_2 foci is overlapped by TDP-43 nuclear aggregates in (A). The 26-kDa TDP-43 fragment hardly accumulates in nuclei and lacks affinity for NEAT1_2 foci. Data represent mean ± s.d. (n = 50 for each transfection of TDP-43 isoforms). *P < 0.0001 (unpaired Student’s *t*-test).

Characteristics of NEAT1_2 lncRNA and paraspeckle formation have been elucidated mainly using cultured cells including HeLa cells [16-20,28-34]. To investigate the cellular basis for the pathogenesis of ALS, we initially examined the subcellular localization of WT, mutant and/or truncated forms of TDP-43 and FUS/TLS by exogenously expressing tagged proteins in HeLa cells. Both WT and the 35-kDa C-terminal fragment of TDP-43 protein were widely distributed throughout the nucleus; however, their characteristic aggregates in the nucleus coincided with 93.8 ± 10.6% and 89.7 ± 13.9% of the NEAT1_2 foci, respectively (Figure 1A, B). These findings mean that almost all NEAT1_2 foci overlap with parts of TDP-43-forming nuclear aggregates. Similar enrichment to NEAT1_2 foci was also observed with tagged WT FUS/TLS (96.9 ± 10.2%; Figure 1A) as well as endogenous TDP-43 and FUS/TLS (Additional file 1: Figure S1A). By contrast, the 26-kDa C-terminal fragment of TDP-43 formed few aggregates in the nucleus, and was distributed throughout both the nucleus and the cytosol, as shown in a previous article [8], demonstrating a marked lack of affinity for NEAT1_2 foci (15.8 ± 17.9%; Figure 1A). Additionally, ALS-associated TDP-43 mutants and FUS/TLS mutants were colocalized with NEAT1_2 lncRNA as frequently as WT TDP-43 and WT FUS/TLS (TDP-43^A315T^: 93.3 ± 12.7%, TDP-43^A382T^: 98.0 ± 8.2%, FUS/TLS^R514S^: 95.8 ± 9.3%, and FUS/TLS^P525L^: 98.4 ± 5.8%; Additional file 1: Figure S1B, C).

The short form of NEAT1 ncRNA, NEAT1_1, is produced from the 5′-end of NEAT1. Although an *in situ* hybridization probe targeting NEAT 1_1 ncRNA, that is shown as NEAT1_1/1_2 probe in Additional file 1: Figure S1D (upper), could not precisely distinguish NEAT1_1 foci from NEAT1_2 foci, most NEAT1_1 foci were also colocalized frequently with nuclear aggregates formed by WT TDP-43 and WT FUS/TLS (Additional file 1: Figure S1D, lower).

**Figure 2:**
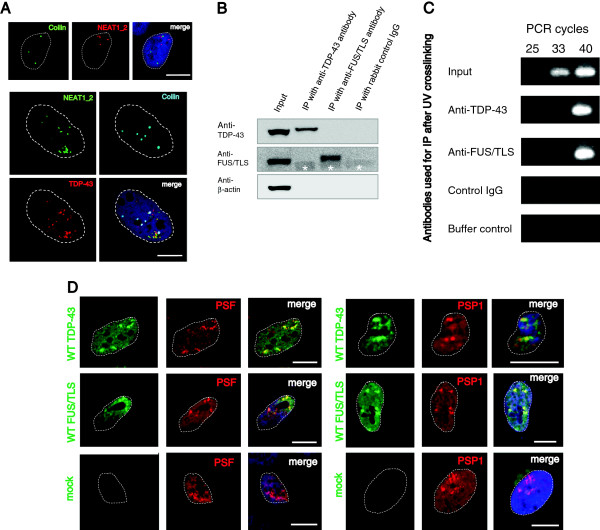
**Both TDP-43 and FUS/TLS bind to NEAT1_2 lncRNA and are colocalized with paraspeckle proteins. A**. Characterization of NEAT1_2 foci and Cajal bodies (marker: coilin). After *in situ* hybridization using DIG-labeled NEAT1_2 probe, untransfected HeLa cells were triple-labeled with monoclonal IgG1 anti-DIG, monoclonal IgG2b anti-coilin and polyclonal anti-TDP-43 antibodies. Upper: NEAT1_2 lncRNA demonstrates a different localization pattern from Cajal bodies. Lower: endogenous TDP-43 overlaps with both NEAT1_2 foci and Cajal bodies. Dotted lines represent the outline of the nucleus. **B**. Using monoclonal antibodies, full-length TDP-43, FUS/TLS and β-actin bands are shown by immunoblotting. Immunoprecipitaion (IP) followed by solid washes in high-salt buffer purified the specific protein against each antibody. Protein-protein interactions were abolished. By combined RNAs, the complexes treated with 254 nm ultraviolet (UV) crosslinking and IP were shifted to the higher molecule compared to the bands of input without UV treatment. *Non-specific detections of the rabbit IgG heavy chains. **C**. NEAT1_2 lncRNA directly binds to TDP-43 and FUS/TLS. Following UV crosslinking in HeLa cells, NEAT1_2 RT-PCR bands are detected with the indicated number of PCR cycles after IP using each antibody. **D**. Immunofluorescence of HeLa cells, which exogenously expressed WT TDP-43 or FUS/TLS with the V5 tag, was carried out at 48 hours after transfection. Monoclonal or polyclonal anti-V5 along with anti-PSF or anti-PSP1 antibodies were used. Dotted lines represent the outline of the nucleus. Scale bars, 10 μm.

Another nuclear body, the Cajal body, is related to RNA metabolism; however, NEAT1_2 foci demonstrated a complete different distribution from Cajal bodies labeled with the marker coilin (Figure 2A, upper). Consistent with a previous report that 40% of TDP-43 nuclear bodies overlapped with Cajal bodies [35], endogenous TDP-43 that did not overlap with NEAT1_2 foci overlapped with the Cajal bodies separately (Figure 2A, lower). In light of TDP-43 and FUS/TLS protein colocalization to NEAT1_2 lncRNA, we tested whether endogenous TDP-43 and FUS/TLS bound directly to NEAT1_2 lncRNA. The RNA-protein complex was immunoprecipitated from UV cross-linked HeLa cells using polyclonal anti-TDP-43 and anti-FUS/TLS antibodies with stringent washes in high-salt buffer to spoil protein-protein interactions. The immunoblotting assay verified specific precipitations by using monoclonal anti-TDP-43 and anti-FUS/TLS antibodies (Figure 2B). After bound RNA was isolated, NEAT1_2 lncRNA levels were quantified by reverse transcription (RT) and polymerase chain reaction (PCR). NEAT1_2 lncRNA was enriched in anti-TDP-43 and anti-FUS/TLS immunoprecipitants compared with control IgG immunoprecipitants (Figure 2C). Paraspeckle formation requires NEAT1_2 lncRNA and core paraspeckle proteins, which subsequently recruit other paraspeckle-associated factors and NEAT1_1 ncRNA [19,29]. Therefore, to determine whether TDP-43 and FUS/TLS formed paraspeckles in cultured cells, immunocytochemistry was performed to examine the intra-nuclear localization of the paraspeckle proteins, PSF and PSP1. Nuclear aggregates of TDP-43 and FUS/TLS colocalized with PSF and PSP1 (Figure 2D). Taking these findings together, NEAT1_2 foci were considered to form paraspeckles with TDP-43 and FUS/TLS.

### NEAT1_2 lncRNA is not expressed in motor neurons in control mouse spinal cord

**Figure 3:**
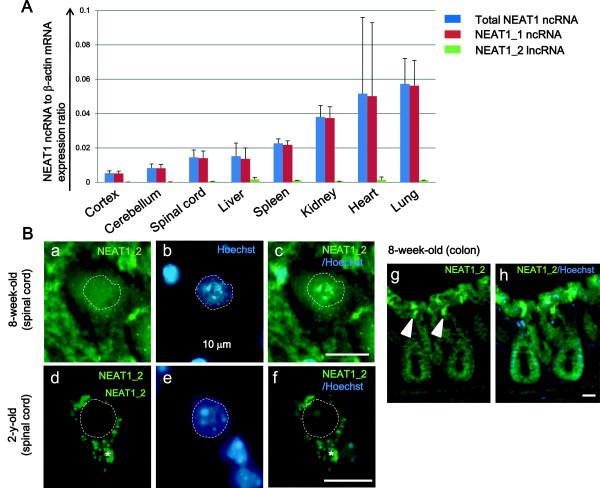
**NEAT1_2 lncRNA is not expressed in the nuclei of WT mouse spinal motor neurons. A**. The expression levels of total NEAT1, NEAT1_1 and NEAT1_2 transcripts in tissues of 8-week-old mice are displayed using quantitative RT-PCR. Data represent mean ± s.d. (n = 3). **B**. NEAT1_2 nuclear lncRNA does not appear in the control mouse spinal cord. (a-c) a motor neuron in 8-week-old mouse spinal cord. (d-f) a motor neuron in 2-y-old mouse spinal cord. The motor neurons in the ventral horn were distinguished from glial cells by their morphological features. Dotted lines represent the outline of the nucleus. Arrowheads: colon epithelial cells served as a positive control for NEAT1_2 detected by *in situ* hybridization (g, h). Lipofuscin in the cytoplasm, which is easily formed in aged motor neurons and has autofluorescence, is denoted as an asterisk (d, f). A long-path filter was used to distinguish Hoechst from autofluorescence. Scale bars, 10 μm.

Next, we examined NEAT1_2 distribution in the nervous system of WT control mice *in vivo*. Some reports have demonstrated that NEAT1_2 expression is abundant in restricted cells including the epithelial cells of the esophagus, forestomach, and surface epithelium of zymogenic region of the stomach in adult mice but not in embryonic stem cells [36]. No previous reports have described NEAT1_2 expression in the spinal cord, aged tissues, or any human tissues. To test the distribution of NEAT1_1 ncRNA and NEAT1_2 lncRNA in each tissue including the nervous system in mice, quantitative RT-PCR was performed using 8-week-old mouse tissue extracts. NEAT1 ncRNA was highly expressed in lung, heart, and kidney (Figure 3A) and markedly dominated by NEAT1_1 expression, consistent with a previous report [36]. Notably, expression levels of NEAT1_1 ncRNA were modest, and NEAT1_2 lncRNA was hardly detectable in the central nervous system. Therefore, we further investigated NEAT1_2 distribution specifically in single spinal motor neurons. *In situ* hybridization followed by fluorescent immunohistochemistry (RNA-FISH) revealed no NEAT1_2 expression in the nuclei of spinal motor neurons from 8-week-old and 2-y-old mice (Figure 3B). NEAT1_1 ncRNA was expressed in the spinal glial cells of both 8-week-old and 2-y-old mice, and was also expressed at low levels in the spinal motor neurons of both young and aged mice (Additional file 2: Figure S2).

### Paraspeckle formation occurs in motor neurons in the spinal cords of human ALS patients

**Figure 4:**
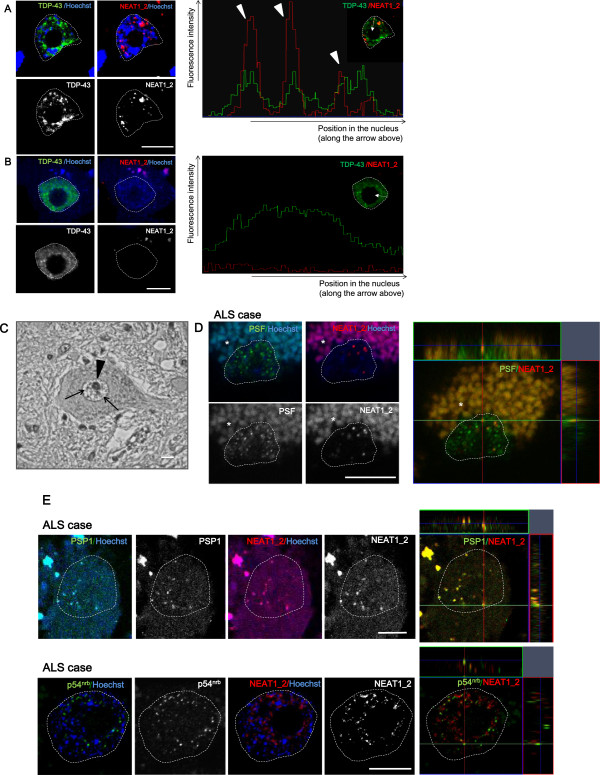
**Paraspeckles appear predominantly in spinal motor neurons in ALS. A**. RNA-FISH using DIG-labeled NEAT1_2 probe indicates that NEAT1_2 lncRNA often appears in the nuclei of human motor neurons in sporadic ALS cases. Right panel shows the profile image of fluorescence intensity of NEAT1_2 lncRNA and TDP-43 along the arrow in the nucleus (ZEN software, Carl Zeiss). Most NEAT1_2 signals overlapped with parts of aggregated TDP-43 in the nucleus (arrowheads). Dotted lines represent the outline of the nucleus. **B**. No NEAT1_2 expression is detected in most motor neurons in control cases. Dotted lines represent the outline of the nucleus. **C**. A 40-fold magnified view of a spinal motor neuron in an ALS case is shown with DAB-hematoxylin counterstain using anti-PSP1 antibody. The arrowhead indicates the nucleolus. Arrows indicate PSP1 aggregates in the nucleus. **D**, **E**. RNA-FISH using DIG- or FITC-labeled NEAT1_2 probe in the nuclei of ALS motor neurons. PSF ((D), ALS-Pt C), PSP1 (upper in (E), ALS-Pt D) and p54^nrb^ (lower in (E), ALS-Pt B) overlap with NEAT1_2 foci. Observation at 647 nm wavelength was performed to rule out autofluorescent signals. Right panels display images on orthogonal sections (ZEN software). Asterisks indicate lipofuscin in the cytoplasm. Dotted line: outline of the nucleus. Scale bars, 10 μm.

While NEAT1_2 lncRNA interacted with ALS-associated proteins in human cell lines (Figure 1A and B, Figure 2C and Additional file 1: Figure S1A), no NEAT1_2 lncRNA was observed in spinal motor neurons in mice *in vivo* (Figure 3B). According to these findings, we hypothesized that NEAT1_2 lncRNA has a particular role in motor neurons in sporadic ALS patients. To test this hypothesis, the fresh frozen spinal cords of six sporadic ALS cases (age: 73.2 ± 8.9 y) and six control cases (age: 83.2 ± 4.4 y) were prepared (Table 1). Contrary to the results in mice (Figure 3B), RNA-FISH demonstrated that more than 80% of human spinal motor neurons in ALS cases displayed NEAT1_2 foci in the nuclei (Figure 4A, 5C). Using sense probe as a negative control, the possibility that antisense probe against NEAT1_2 lncRNA recognized other non-specific intranuclear RNAs was excluded in the human spinal cord (Additional file 3 Figure S3). Parts of endogenous TDP-43 aggregates in the nucleus coincided with nearly all NEAT1_2 foci in motor neurons in ALS cases (Figure 4A), consistent with findings in cultured cells (Figure 1A and B, Figure 2C and Additional file 1 Figure S1A).

**Table 1:** Profiles of individual ALS and control cases in this study

**Patient**	**Sex/Age (y)**	**Disease**	**Duration of illness (months)**	**Duration on respirator** (**months**)	**TDP-43 cytoplasmic aggregation in spinal motor neurons**	**Cause of death**	**Postmortem delay until resection (min)**	**Number of spinal motor neurons examined in this study**
**A**	**Female** / **66**	**sporadic ALS**	**38**	**26**	+	**Pneumonitis**	**82**	**74**
**B**	**Male** / **59**	**sporadic ALS**	**45**	**26**	+	**empyema**	**113**	**104**
**C**	**Female** / **76**	**sporadic ALS**	**39**	**18**	+	**pneumonitis**	**127**	**93**
**D**	**Male** / **76**	**sporadic ALS**	**12**	-	+	**pneumonitis**	**112**	**92**
**E**	**Male** / **83**	**sporadic ALS**	**27**	-	+	**pneumonitis**	**123**	**143**
**F**	**Female** / **79**	**sporadic ALS**	**31**	-	+	**pneumonitis**	**240**	**127**
**C1**	**Female** / **79**	**acute myocardial infarction**	-	-	-		**97**	**64**
**C2**	**Male** / **87**	**Alzheimer**’**s disease**, **lung cancer**, **hypertension**	-	-	-		**240**	**47**
**C3**	**Female** / **86**	**dementia**, **acute myocardial infarction**, **hypertension**	-	-	-		**240**	**43**
**C4**	**Female** / **81**	**colon cancer**, **post**-**cerebral infarction**	-	-	-		**128**	**36**
**C5**	**Male** / **78**	**metastatic brain tumor**	-	-	-		**103**	**100**
**C6**	**Male** / **88**	**dementia**	-	-	-		**250**	**49**

The average ages in ALS and control cases were 73.2 ± 8.9 y and 83.2 ± 4.4 y, respectively. All ALS cases in this study showed TDP-43 cytoplasmic aggregation in the spinal cord. Age, age of the patient at death; Patient A-F, individuals with ALS; C1-C5, normal controls with no neurological disorder affecting the spinal cord.

**Figure 5:**
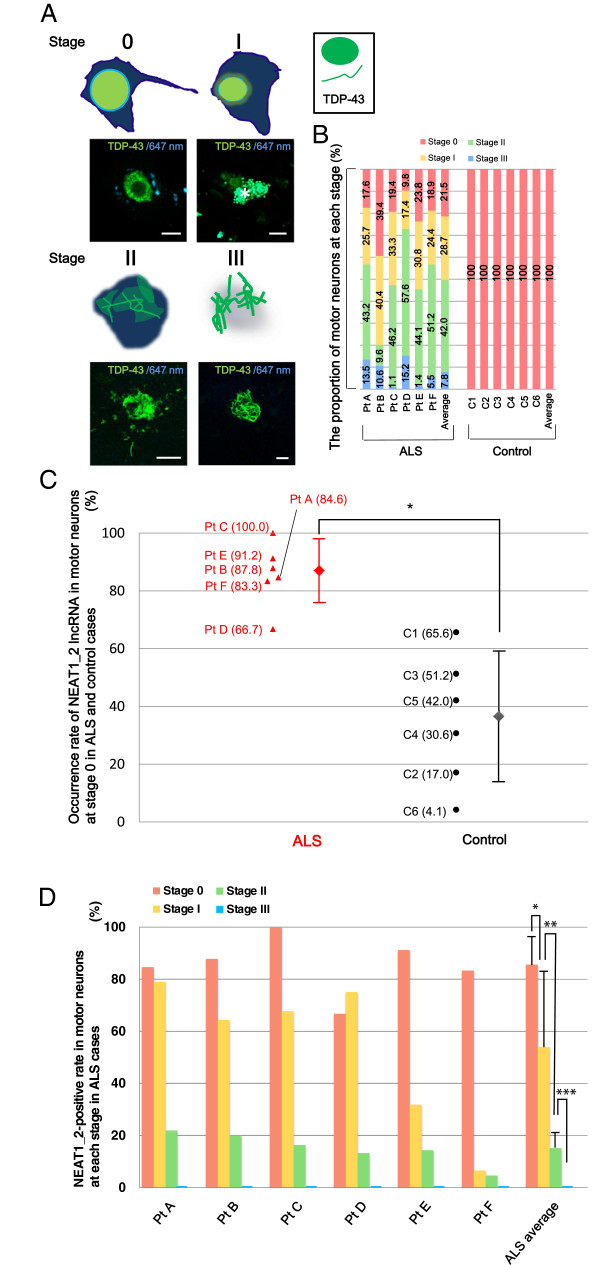
**Paraspeckle formation is frequently observed in an early phase of ALS. A**. A scheme of TDP-43 aggregation patterns in ALS is presented. Observations detected at 647 nm are added to distinguish the TDP-43-positive signals from autofluorescence produced by lipofuscin granules in the cytoplasm (shown as an asterisk). Scale bars, 10 μm. **B**. Stages of motor neurons in sporadic ALS and control cases are shown according to the definitions in Table 2. The number on the bar graph represents the proportion of motor neurons at each stage in a case. **C**. NEAT1_2-positive rates in the spinal motor neurons at stage 0 are indicated (ALS vs. control). Each triangle or circle represents an individual ALS (Pt) or control (C) patient, respectively. The actual value of percentage in each case is shown in parenthesis. Bars represent the mean ± s.d.; that is, 85.6 ± 11.0% in ALS cases and 35.1 ± 22.6% in control cases (n = 6 per group). *P<0.001. **D**. NEAT1_2-positive rates in spinal motor neurons at each stage are plotted. Mean ± s.d. were 54.0 ± 28.6% at stage I, 15.1 ± 6.1% at stage II, and 0.0 ± 0.0% at stage III (n = 6 per group). *P<0.05, **P<0.01, ***P<0.001.

Next, to test whether NEAT1_2 lncRNA in human motor neurons formed paraspeckle structure, the nuclear distribution pattern of PSP1 was examined with immunohistochemistry and visualized with DAB (Figure 4C). PSP1 was often observed as an aggregated form in the nuclei of motor neurons as well as surrounding glial cells. In addition, RNA-FISH demonstrated that PSF, PSP1, and p54^nrb^ were colocalized with NEAT1_2 foci in the nuclei of ALS motor neurons (Figure 4D and E, and Additional file 4: Figure S4). In control cases, more than 60% of motor neurons demonstrated no NEAT1_2 foci (Figure 4B, 5C); however, the remaining motor neurons contained NEAT1_2 foci with paraspeckle proteins in the nuclei (Additional file 4: Figure S4). These results suggest that paraspeckle proteins have affinity for NEAT1_2 foci in motor neurons in both ALS and control cases.

### Paraspeckles appear predominantly in spinal motor neurons in the early phase of the pathological process

To test whether paraspeckles in the motor neuron were formed significantly in sporadic ALS, occurrence rates of NEAT1_2 lncRNA in human motor neurons were quantified. All motor neurons were regarded as the subjects of the number count in the ventral horns of several spinal cord slices at a constant thickness of 14 μm. The motor neurons were definitively-distinguishable from surrounding glia cells according to the morphology, specific structures including lipofuscin, or the size that is more than approximately 35 μm. To evaluate occurrence rates of NEAT1_2 lncRNA at the same stage of TDP-43 distribution, we subdivided the pathological stages of ALS spinal motor neurons into four classes (Table 2, Figure 5A). At Stage 0, TDP-43 is normally distributed within the nucleus. Since TDP-43 is seen in the cytoplasm or the nucleus of the motor neuron degrades, stage I, II and III indicate pathological stages in ALS. The nucleus degrades but is still recognized at stage I. In stage II, the nuclear TDP-43 was not recognized any more, however, the plasma membrane seems still retained. Stage III is the final stage, where the plasma membrane disappeared and TDP-43 skein-like inclusions are left behind. ALS cases demonstrated motor neurons at all pathological stages, in contrast to control cases in which all motor neurons were limited to stage 0 (Figure 5B). In overall counting of the motor neurons in the ventral horn, there was no significant difference in the NEAT1_2 lncRNA appearance rate between in ALS and control cases (Additional file 5: Figure S5). Only at stage 0, however, the occurrence of NEAT1_2 expression in motor neurons was significantly increased in ALS cases compared to that in control cases (85.6 ± 11.0% and 35.1 ± 22.6%, respectively; Figure 5C). The positive rate of NEAT1_2 foci in motor neurons was higher at the early stage of ALS than at the advanced stage (Figure 5D). This finding suggests that NEAT1_2 lncRNA appeared preferentially in spinal motor neurons at the earliest point of ALS.

**Table 2:** **Pathological staging of motor neurons in ALS according to TDP**-**43 distribution**

**Stage 0**	**TDP**-**43 is normally distributed within the well**-**marginated nucleus**.			
**Stage I**	**The nucleus degradated**, **and TDP**-**43 was also seen in the cytoplasm**.			
**Stage II**	**The nuclear TDP**-**43 was so cleared that it was not recognized**.**The plasma membrane was still retained**.			
**Stage III**	**The plasma membrane disappeared**.			
	TDP-43 distribution		nuclear membrane	plasma membrane
	in the nucleus	in the cytoplasm		
Stage 0	+	-	+	+
Stage I	+	+	±	+
Stage II	ND	+	-	+
Stage III	ND	ND	-	-

ND, not determined.

### Characterization of nuclear paraspeckles by electron microscopy

**Figure 6:**
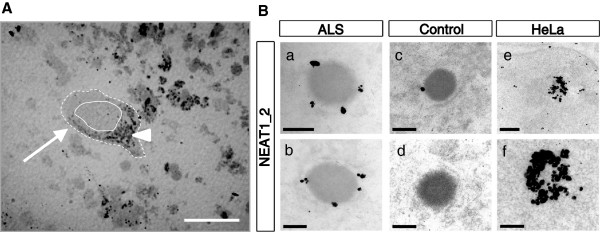
**NEAT1_2 lncRNA aggregates around the IGAZ forming paraspeckles in motor neurons of the ALS spinal cord. A**. Semi-thin sections (0.5 μm thickness) were observed with toluidine blue staining to identify a motor neuron (arrow), which characteristically includes lipofuscin (arrowhead) in the cytoplasm. Solid line, outline of the nucleus; dotted line, outline of the cytoplasm. Scale bar, 50 μm. **B**. Electron microscopic observation after *in situ* hybridization using DIG-labeled RNA probe targeting NEAT1_2 lncRNA in the nucleus of a motor neuron in the spinal cord of an ALS patient (Pt B). The interchromatin granule-associated zone (IGAZ) or IGAZ-like zone is observed as an intermediate electron-dense cluster. The gold-labeled probe visualizes NEAT1_2 lncRNA as a halo-shaped pattern of aggregations around IGAZ in the nucleus of an ALS spinal motor neuron (a, b). A control case (C3) demonstrated few NEAT1_2 granules around the IGAZ and/or IGAZ-like zone (c, d). Observation in HeLa cells is shown as a positive control for the formation of paraspeckle structures (e, f). Scale bars, 200 nm (a-d, f) and 500 nm (e).

The nuclear distribution pattern of NEAT1_2 lncRNA in the human nervous system has not been previously reported, and its function remains largely unknown. To observe NEAT1_2 localization in human spinal motor neurons in detail, electron microscopic analysis combined with *in situ* hybridization (EM-ISH) was performed. In the spinal cords of human ALS cases, NEAT1_2 lncRNA was localized to IGAZ in a halo-like fashion, suggesting that NEAT1_2 lncRNA formed the characteristic structure of paraspeckles in the nuclei of spinal motor neurons in ALS cases (Figure 6B-a and -b, and Additional file 6: Figure S6C), consistent with previous articles [17,28,30]. By contrast, NEAT1_2-labeled gold particles around IGAZ were rare in control cases (Figure 6B-c and -d). The IGAZ has been considered to be identical to a paraspeckle [17,28-30], indicating that the formation of IGAZ depends on NEAT1_2 lncRNA. IGAZ-like zone without NEAT1_2 granules in Figure 6B-d suggests that a human motor neuron has a moderately electron-dense intranuclear structure besides paraspeckles. Alternatively, independent of NEAT1_2 lncRNA, IGAZ may exist in the nucleus as a scaffold prior to the recruitment of NEAT1_2 lncRNA to form a complete paraspeckle. NEAT1_1/1_2 probe, detecting both NEAT1_1 ncRNA and NEAT1_2 lncRNA (Additional file 1: Figure S1D, upper), was observed in the nuclei of spinal motor neurons in control cases as frequently as those in ALS cases (Additional file 6: Figure S6A-a, -b, -c and -d). Notably, halo-shaped aggregation patterns formed by NEAT1_1/1_2 ncRNAs were found throughout nuclei in ALS spinal motor neurons (Additional file 6: Figure S6A-b) but were observed rarely in control cases (Additional file 6: Figure S6A-c and -d). Even when diluted NEAT1_1/1_2 probe is used, both central (Additional file 6: Figure S6B-a) and halo-like (Additional file 6: Figure S6B-b) patterns of aggregation were observed in a HeLa cell. This suggests that NEAT1_1 ncRNA may show the central accumulation pattern independently of the IGAZ margin; however, it could demonstrate the halo-shaped pattern depending on NEAT1_2 lncRNA. Taken these findings together, NEAT1_2 lncRNA forms paraspeckles specifically around IGAZ in the nuclei of motor neurons in ALS cases, which may affect the distribution of NEAT1_1 ncRNA.

## Discussion

In this study, we demonstrated that a lncRNA with GC-rich sequence, NEAT1_2, is predominantly expressed in spinal motor neurons in an early phase of the ALS pathological process. To our knowledge, this report is the first to indicate a direct association of paraspeckle formation with a human neurodegenerative disease.

The involvement of abnormalities in functional RNAs has been reported in the development of various neurodegenerative disorders. For example, (CAG)n triplet repeats encoding polyglutamine induce spinocerebellar ataxia type 2; however, when glutamine-coding sequences (CAA)s are inserted into the CAG repeats, this genotype becomes a risk factor for ALS [37]. Moreover, (GGGGCC)n hexanucleotide repeat expansion in ALS has been described in the intron of the C9ORF72 gene recently. Any ALS cases in the present study did not have (GGGGCC) hexanucleotide repeat expansion, but previous report showed that RNA consisting of GGGGCC-expanded repeats formed characteristic foci in the nuclei of human spinal motor neurons [3]. These findings suggest that alteration in RNA metabolism may be important for the ALS pathomechanism in specific nuclear foci including RNAs with the GC-rich sequence.

Full-length and 35-kDa TDP-43 were retained in NEAT1_2 foci (Figure 1A, B, and Additional file 1: Figure S1A), suggesting that the appearance of NEAT1_2 lncRNA may change functions of these TDP-43 proteins to process RNAs and to form stress granules. By contrast, NEAT1_2 foci did not retain the 26-kDa fragment of TDP-43 (Figure 1A, B). Even when NEAT1_2 lncRNA appears in the nucleus, insoluble 26-kDa TDP-43 with phosphorylation is mislocalized to the cytoplasm, consistent with previous pathological findings [12].

The direct binding of TDP-43 or FUS/TLS to NEAT1_2 lncRNA was verified in Figure 2C. In a recent article, iCLIP data targeting TDP-43 suggested that NEAT1_2 lncRNA was one of the target RNAs bound predominantly by TDP-43 in human brain tissue with TDP-43 proteinopathy and cultured cells [23]. According to this report, 5% of TDP-43 iCLIPed cDNA was mapped to lncRNA regions, and TDP-43 demonstrated high binding affinities to UG-repeat clusters in positions 6,662–6,728 and 21,464–21,544 of NEAT1_2 lncRNA (Additional file 7: Figure S7). Additionally, another recent report presented proteomic evidence for TDP-43 co-aggregation with several paraspeckle proteins, suggesting that paraspeckle formation may have a strong influence on the localization and function of TDP-43 in the nucleus [38].

Similarly, previous reports indicated that NEAT1_2 lncRNA is one of the RNAs directly bound by FUS/TLS. Wang et al. demonstrated that FUS/TLS preferentially interacts with GGUG RNA oligonucleotides [24]. Another recent report using iCLIP methods in mouse brain also revealed that the GGU motif increases the affinity of FUS/TLS for RNA [25]. Using mouse and human brain, the latest CLIP-seq data showed enrichment of the GUGGU motif in FUS/TLS clusters [26]. These reports suggest that UG-rich sequences are the preferred binding sites of both TDP-43 and FUS/TLS; however, Rogelj et al. did not mention any overlap between the sequence specificity of TDP-43 and FUS/TLS [25]. By contrast, PAR-CLIP procedure revealed that the FUS/TLS-binding sites frequently involve the *SON* cluster or AU-rich stem-loops, unlike the previous reports described above [27]. This procedure revealed NEAT1 ncRNA to be a target of both WT and mutant FUS/TLS. Interestingly, one more binding site in NEAT1 ncRNA was identified using mutant FUS/TLS than using WT FUS/TLS, suggesting the possibility that the affinity or binding pattern of mutant FUS/TLS may differ from that of WT FUS/TLS [27]. Here, we found that the NEAT1_2 sequence possessed at least three similar stem-loops to the *SON* cluster that was proposed as a FUS/TLS-binding site (Additional file 7: Figure S7). In particular, the most upstream cluster, which starts from the nucleotide at position 3,440, is highly AU-rich in content, suggesting that FUS/TLS binds most preferably to this site of NEAT1_2 lncRNA.

Importantly, the frequency of paraspeckle formation increased significantly during the early phase of ALS pathological course (Figure 5C, D). The possibility that aging simply induced an increase in the level of NEAT1_2 lncRNA was eliminated based on two observations. First, NEAT1_2 lncRNA did not appear in the spinal motor neurons of 2-y-old control mice (Figure 3B), and second, human control cases in this study were older than ALS cases by an average of 10 y (Table 1). The ventral spinal motor neurons include α- and γ-motor neurons besides interneurons. Here, we counted all spinal motor neurons on the slides. In the normal condition, α-motor neurons can be distinguished definitely from γ-motor neurons and interneurons by the size; that is, α- for more than 35 μm and γ- and interneurons for less than 35 μm. In this study, however, the α-motor neurons had degenerated to get smaller, looking like γ-motor neurons or interneurons. To the best of our knowledge, any reliable molecular marker has never been verified to stain α-motor neurons specifically in the human tissue. Therefore, we could not investigate the difference of NEAT1_2 lncRNA occurrence according to the type of neurons in the spinal cord. Developing the specific human marker of the α-motor neuron, further investigations are required to determine this point.

This would be also among the first reports to show EM-ISH images focusing on the nuclei of human motor neurons. We confirmed that the NEAT1_2 foci in motor neurons demonstrated characteristics of paraspeckle formation, in which the NEAT1_2 probe revealed a halo-like appearance around IGAZs (Figure 4C–E and Figure 6B). By contrast, the NEAT1_1/1_2 probe often demonstrated a central accumulation pattern, but occasionally formed the halo-like pattern in the presence of NEAT1_2 lncRNA (Additional file 6: Figure S6A, B). These results indicate that NEAT1_2 lncRNA may influence NEAT1_1 ncRNA localization and possibly function.

As reported previously, *Lin28* mRNA transcript and cationic amino acid transporter 2 transcribed nuclear RNA with hyper-edited sites are retained in paraspeckles [15,16], suggesting that RNA editing alterations may dynamically change cell fate by retaining RNA in paraspeckles. Insufficient GluA2 mRNA (Q/R-)editing efficiencies in motor neurons were identified specifically in sporadic ALS cases [14]. Translation of a small amount of the edited GluA2 mRNA in ALS could potentially be suppressed through paraspeckle formation, while unedited GluA2 mRNA is perhaps predominantly released from nuclear paraspeckles into the cytoplasm, causing toxicity to motor neurons.

Both TDP-43 and FUS/TLS are paraspeckle proteins, which are required for normal paraspeckle formation through the direct interactions with NEAT1_2 lncRNA. According to the previous report using cultured cells, the number and size of paraspeckles were decreased significantly by TDP-43 RNAi, and paraspeckle formation was completely disrupted under the FUS/TLS knockdown [33]. FUS/TLS also associates with RNA polymerase II, which is another paraspeckle protein [39,40]. RNA polymerase II is not only responsible for polyadenylation, but also for determination of the 3′-endpoint in pre-mRNA processing in cooperation with other paraspeckle proteins, PSF and p54^nrb^[41,42]. Paraspeckle proteins could induce processing of target RNAs bound to FUS/TLS or TDP-43 in ALS spinal motor neurons [43]. The components of the paraspeckle, including RNA polymerase II, PSF and p54^nrb^, may produce RNAs with their 3’UTRs truncated, which could be resistant to microRNAs. Screening of the target RNAs assembled in the paraspeckles will be highly informative. In particular, identification of 3′-end sites or alternative splicing patterns of target RNAs may be key for future investigations.

**Figure 7:**
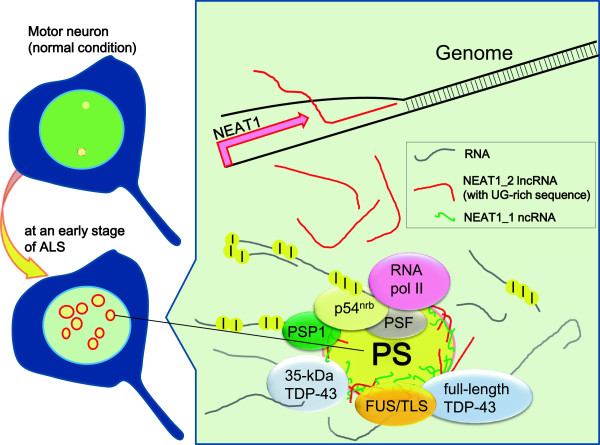
**Scheme of the pathological alterations in the nucleus of an ALS motor neuron.** NEAT1_2 expression is induced by ALS-associated mechanisms. NEAT1_2 lncRNA forms paraspeckle structure around IGAZ with NEAT1_1 RNA, RNA-binding proteins consisting of TDP-43 and FUS/TLS, and other paraspeckle proteins including p54^nrb^, PSF, PSP1 and RNA polymerase II. The right panel represents enlarged illustration of the nucleus of a motor neuron at an early stage of ALS. PS, paraspeckle; I, inosine at an A-to-I RNA editing site.

The summarized scheme at an early phase of ALS is shown in Figure 7. Certain stresses may cause NEAT1_2 lncRNA-induced paraspeckle formation in the nucleus, however, it still remains unknown what mechanism is involved in the upregulation of NEAT1_2 lncRNA during early phase of ALS.

The recent reports have described lncRNA could be subject to epigenetic regulation, especially histone methylation, depending on cell types; thus, investigation of the distribution and levels of H3K4 and H3K27 methylation is required to understand regulation mechanism of NEAT1_2 lncRNA in the future [44]. The paraspeckle retains functional RNA-binding proteins including TDP-43, FUS/TLS, and the other paraspeckle proteins, culminating in an ectopic structure that may serve as a platform for RNA metabolism associated with ALS. Hyper-edited RNAs and/or RNAs preferably bound to paraspeckle component proteins may be captured in paraspeckles and processed aberrantly prior to export to the cytoplasm. There is another possibility that paraspeckles with the NEAT1_2 lncRNAs appear under certain stressful condition during early phase of ALS and alleviate the toxicity by regulating the specific RNA splicing events and/or the 3’UTR determinations. As the pathological stage progresses, neuronal degeneration might occur with the decrease of paraspeckles.

Screening of the target RNAs assembled in the paraspeckles with RNA-binding proteins will also be highly informative. In particular, identification of 3′-end position or alternative splicing patterns of target RNAs and regulation in microRNA biogenesis may be key for future investigations as described above. Success in this screening largely depends on technological innovation in the field of CLIP and RNA sequencing [27,45,46].

For the next step, another investigation into changes of phenotype after crossing ALS model mice with NEAT1_2 knockout mice is required to verify, at least in part, whether the increase in NEAT1_2 lncRNA has a protective or damaging role in the ALS pathological pathway. Additionally, NEAT1_2 knock-in mice and human induced pluripotent stem cells-derived motor neurons under exogenous NEAT1_2 expression should be characterized.

In conclusion, this study identified the ‘paraspeckle’ formation containing a nuclear lncRNA, NEAT1_2, in the early phase of ALS, shedding light on novel therapeutics for motor neuron degeneration.

## Materials and methods

### Cell culture and reagents

HeLa human carcinoma cells were maintained in Dulbecco’s modified Eagle’s medium (Gibco, Grand Island, NY) supplemented with 10% fetal bovine serum as described previously [8,47]. Transfection was performed using GeneJuice Transfection Reagent (Novagen, Madison, WI) according to the manufacturer’s instructions. To transfect HeLa cells, cells were grown to 60–80% confluence on 0.001% poly-L-lysine (PLL)-coated coverslips or 8-chamber slides (Chamber slide II, Iwaki Glass, Tokyo, Japan). Forty-eight hours after transfection, cells were fixed in 4% paraformaldehyde (PFA) overnight at 4°C for *in situ* hybridization or for 10 min at room temperature for immunofluorescence staining. Cells were washed with phosphate-buffered saline (PBS), and total RNA was extracted using TRIzol reagent (Invitrogen, Carlsbad, CA) for quantitative RT-PCR.

### Human tissue frozen samples and animal tissue samples

Human spinal cords were stored at -80°C immediately after removal from the body. Informed consent was given by the legal guardians of the patients and all experimental procedures in this study were carried out in accordance with the Declaration of Helsinki principles and with the approval of the Ethics Committees of School of Medicine, Keio University (no. 20100268), Tokyo Metropolitan Geriatric Hospital (no. 73), RIKEN Advanced Science Institute (no. 23-3), and Institute of Brain and Blood Vessels, Mihara Memorial Hospital (no. 049-01). To quantify RNA expression levels, 8-week-old C57BL/6 mice were anesthetized with pentobarbital and perfused with PBS, and then each tissue was eluted in TRIzol followed by RNA extraction and quantitative RT-PCR. For staining, perfusion was performed with 4% PFA in PBS. All animal experiments were conducted in accordance with an animal protocol approved by the Laboratory Animal Care and Use Committee of Keio University. Tissues were then resected for cryoprotection with 20% sucrose in PBS and embedded in Tissue-Tek (Sakura Finetek, Torrance, CA). Sections of human or mouse dissected tissues (14 μm thick) on PLL-coated glass slides (Matsunami Glass, Osaka, Japan) were dried and refixed in 4% PFA overnight at 4°C, followed by immunofluorescence staining, *in situ* hybridization, or immunohistochemistry as described below.

### Antibodies

Among primary antibodies, mouse monoclonal anti-V5 (1:500, R960-25) was purchased from Invitrogen. Rabbit polyclonal anti-V5 (1:500, A190-120A) and rabbit polyclonal anti-FUS/TLS (1:250, A300-302A) were from Bethyl Labs (Montgomery, TX). Rabbit polyclonal anti-TDP-43 (1:250, 10782-2-AP) was from Proteintech (Chicago, IL). These polyclonal antibodies directed against FUS/TLS and TDP-43 were used for immunochemistry and immunoprecipitation. Mouse monoclonal antibodies, anti-TDP-43 (1:1,000, H00023435-M01) from Abnova (Walnut, CA) and anti-FUS/TLS (1:1,000, 4H11, sc-47711) from Santa Cruz Biotechnology (Santa Cruz, CA), were used for immunoblotting. Rabbit polyclonal anti-fluorescein isothiocyanate (FITC) (1:500, ab19491), mouse monoclonal anti-digoxigenin (DIG) IgG1 (1:500, H8, ab420), and mouse monoclonal anti-coilin IgG2b (1:500, IH10, ab87913) were from Abcam (Cambridge, MA). Mouse monoclonal anti-β-actin (1:10,000, AC-15, A1978) and anti-PSF (1:200, B92, P2860) antibodies were from Sigma (St. Louis, MO). Mouse monoclonal anti-p54^nrb^ (1:200, 3/p54nrb, 611279) was from BD Transduction Laboratories (San Diego, CA). Rabbit polyclonal anti-PSP1 (1:500) was generated by T. H. The secondary antibodies used for immunofluorescence, Alexa Fluor 488 goat anti-rabbit IgG (A-11034), Alexa Fluor 488 goat anti-mouse IgG1 (A-21121), Alexa Fluor 488 goat anti-mouse IgG2b (A-21141), Alexa Fluor 555 goat anti-rabbit IgG (A-21429), Alexa Fluor 555 goat anti-mouse IgG (A-21424), Alexa Fluor 555 goat anti-mouse IgG1 (A-21127), and Alexa Fluor 647 goat anti-mouse IgG2b (A-21242), were purchased from Life Technologies (Carlsbad, CA).

### Vectors

Plasmids containing the V5-tagged series of TDP-43 and FUS/TLS were kindly provided by Dr. Daisuke Ito (Keio University) [8,9].

### RNA-FISH

Fixed cells or tissues on PLL-coated glass slides were treated with 0.2 N HCl for 20 min, followed by 3 μg/ml proteinase K (PCR grade; Roche, Indianapolis, IN) at 37°C for 3 min (in the case of cell samples) or 7 min (in the case of tissue samples). After acetylation in a solution consisting of 1.5% triethanolamine, 0.25% acetic anhydride, and 0.25% HCl, the hybridization reaction was carried out with DIG- or FITC-labeled probes (1 μg/ml) for 16 h at 55°C in hybridization buffer (50% formamide, 2× SSC, 1× Denhardt’s solution, 5% dextran sulfate, 10 mM ethylenediaminetetraacetic acid (EDTA), and 0.01% Tween 20). Samples were washed twice at 55°C for 30 min with a solution consisting of 50% formamide, 2× SSC, and 0.01% Tween 20, and then 10 μg/ml RNase A was added at 37°C for 1 h in buffer (0.5 M NaCl, 10 mM Tris (pH 8.0), 1 mM EDTA, and 0.01% Tween 20). They were washed in 2× SSC wash buffer with 0.01% Tween 20 at 55°C for 30 min, followed by washing in 0.2× SSC wash buffer with 0.01% Tween 20 at 55°C for 30 min. After additional washing in Tris-buffered saline (TBS, pH 7.6) and blocking in a buffer consisting of blocking reagent (Roche 11096176001), 0.1 M maleate, 0.15 M NaCl, and 0.01% Tween 20 in TBS, DIG- or FITC-labeled probes were detected by standard immunohisto-/immunocytochemical procedures using antibodies against DIG, FITC, and other proteins.

In preparation for DIG- or FITC-labeled RNA probes, cDNA fragments were amplified using M13 forward (FW) and reverse (RV) primers with the AV089414 EST clone as a template for the mouse NEAT1_1/1_2 probe. cDNA fragments were subcloned into pCRII (Invitrogen) for the mouse NEAT1_2 probe after amplification using mNEAT1_2 FW/RV primers with the BAC clone RP23-209P9 as a template [36]. For the human NEAT1_1/1_2 probe and NEAT1_2 probe, cDNA fragments were obtained using hNEAT1 FW/RV primers and hNEAT1_2 FW/RV primers, respectively, using a cDNA library derived from HeLa cells as a template and were subcloned into pCRII-TOPO (Invitrogen) and pGEM-T Easy vectors (Promega, Madison, WI). Each primer is shown in Additional file 8: Table S1. Both antisense and negative control sense RNA probes were prepared using a DIG/FITC RNA labeling mix (1277073/1685619, Roche), RNasin Plus RNase Inhibitor (N2611, Promega), and T3, T7 or SP6 RNA polymerase (P2083/ P2075/P1085, respectively, Promega) according to the manufacturer’s instructions. The usability of each probe was finally characterized by Northern blot.

### CLIP assay

CLIP assay was performed as described previously [48,49]. Briefly, HeLa cells were UV cross-linked at 254 nm (UV-B) with 600 J/cm^2^ in a UV Stratalinker 1800 crosslinker (Stratagene, La Jolla, CA); lysed in wash buffer containing 1× PBS, 0.1% sodium dodecyl sulfate (SDS), 0.5% deoxycholate, and 0.5% NP-40; supplemented with 0.015 U/μl RNasein Plus (N261, Promega) and RQ RNase-Free DNase (M610A, Promega); and immunoprecipitated for 2 h at 4°C with 5 μg polyclonal anti-TDP-43, anti-FUS/TLS, and rabbit IgG control (AB-105-C, R&D Systems, Minneapolis, MN) antibodies bound in advance to Dynabeads Protein G (100.04 D, Invitrogen). Immunoprecipitated materials were washed as follows: twice with wash buffer described above for 5 min; twice with 5× PBS, 0.1% SDS, 0.5% deoxycholate, and 0.5% NP-40 for 5 min (using high-salt wash buffer to completely remove indirect protein-RNA interactions); and twice with 50 mM Tris-HCl (pH 7.4), 10 mM MgCl_2_, and 0.5% NP-40 for 5 min. The immunoprecipitated protein-bead complexes were removed from RNA by proteinase K (13731196, Roche) digestion. RNA was then isolated by phenol/chloroform extraction and treated again with DNase I before the RT reaction was performed as described below. Bound RNAs were evaluated by PCR assay using the qhNEAT1_2 FW/RV primers listed in Additional file 8: Table S1. The PCR reaction was performed in a 50 μl reaction mixture containing 0.2 μM of each primer, 0.25 mM dNTP mix (Takara, Otsu, Japan), 5 μl 10× PCR buffer, and 1 μl Advantage 2 Polymerase mix (639201, Clontech, Mountain View, CA). PCR amplification began with a denaturation step at 95°C for 1 min, followed by 25, 33, or 40 cycles of denaturation at 95°C for 10 s, annealing at 64°C for 30 s, and extension at 68°C for 30 s.

### Immunoblotting

Cells and UV cross-linked immunoprecipitates after stringent washes with the high-salt wash buffer were lysed in cold lysis buffer (50mM Tris–HCl, pH 7.4, 150 mM NaCl, 0.5% Nonidet P-40, 0.5% sodium deoxycholate, 0.25% SDS, 5mM EDTA and Complete, EDTA-free Protease Inhibitor Cocktail Tablets (1873580, Roche)). The input cell lysate was briefly sonicated and lysed samples were separated via reducing SDS-PAGE on a 4–20% Tris-glycine gradient gel (Invitrogen). Proteins were then transferred onto a polyvinylidene difluoride membrane (Millipore, Billerica, MA). The membrane was incubated with primary antibodies, followed by horseradish peroxidase-conjugated secondary antibodies. Proteins were visualized using electrochemiluminescence (ECL) detection reagent (GE Healthcare, Milwaukee, WI) and an ImageQuant LAS 4000 digital imaging system (GE Healthcare).

### Immunofluorescence

Fixed cells or tissues were permeabilized in 0.2% Triton X-100 for 10 min. After blocking for nonspecific binding in TNB blocking buffer (NEL702, Perkin Elmer, Norwalk, CT), samples were incubated with primary antibodies, washed three times in PBS with 0.1% Tween-20, and incubated with secondary antibodies described above. Immunofluorescent images were obtained using a confocal microscope LSM700 (Carl Zeiss, Oberkochen, Germany).

### Immunohistochemistry

Paraffin-embedded tissue sections were deparaffinized, pretreated with 0.3% H_2_O_2_ for 30 min, boiled in citrate solution (pH 6.0), and reacted with TNB blocking buffer, anti-PSP1 primary antibody, and biotin-conjugated donkey anti-rabbit IgG secondary antibody (711-066-152, Jackson Immunoresearch, West Grove, PA). Signals were enhanced with the Vectastain ABC kit (Vector Laboratories, Burlingame, CA). Visualization was performed with DAB (Wako, Osaka, Japan) and 1:20,000 dilution of saturated H_2_O_2_ to observe PSP1 immunopositivity in the nucleus of the motor neuron. The figures were examined using AxioVision imaging software (Carl Zeiss).

### Quantitative RT-PCR

Total RNA was extracted from TRIzol reagent-treated samples using an RNAspin Mini RNA Isolation kit (74106, Qiagen, Germantown, MD) after chloroform/phenol extraction [14]. After treatment with DNase I, RT was carried out using Ready-To-Go You-Prime First-Strand Beads (27-9264-01, GE Healthcare) according to the manufacturer’s instructions. The resultant cDNA was measured by quantitative PCR with SYBR Premix Ex Taq (Perfect Real Time) (RR041A, Takara) in the Mx3000p system (Stratagene) using Rox as a reference and the following primers: qmNEAT1 FW/RV for mouse NEAT1_1/1_2 cDNA, qmNEAT1_2 FW/RV for mouse NEAT1_2 cDNA, and qmActin FW/RV for mouse β-actin cDNA. All primer sequences are listed in Additional file 8: Table S1. Primers for PCR to detect NEAT1_1 cDNA alone could not be designed; thus, the amount of NEAT1_1 expression was calculated by subtracting the copy number of NEAT 1_2 cDNA from that of total NEAT1 cDNA. The reaction was performed at 95°C for 10 min, followed by 50 cycles at 95°C for 30 s, 60°C for 1 min, and 72°C for 30 s after serial dilutions ranging from 10^10^, 10^8^, 10^6^, 10^4^, 10^2^, to 10^1^ copies per 1 μl of DNA solution were prepared as standard samples by subcloning each PCR product into the Zero Blunt TOPO PCR Cloning Kit (K2800, Invitrogen).

### EM-ISH

For transmission electron microscopic analysis, frozen sections of the human spinal cord and HeLa cells were used. As mentioned above, *in situ* hybridization with DIG-labeled RNA probes targeting NEAT1_2 or NEAT1_1/1_2 was performed, which included proteinase K digestion at 37°C for 5 min for human tissue sections and 50 s for HeLa cells. Samples were incubated for 72 h at 4°C with mouse anti-DIG (1:250) and rabbit anti-PSP1 (1:250) primary antibodies. Following washes in 0.005% saponin containing 0.1 M phosphate buffer (PB) for 2 h, samples were incubated for 24 h at 4°C with fluorescence- and Nanogold-conjugated anti-mouse secondary antibodies (1:100, Life Technologies) along with fluorescence-conjugated anti-rabbit secondary antibodies (1:800, Life Technologies). After another wash with PB, samples were observed with LSM700. Samples were fixed with 2.5% glutaraldehyde for 10 min at 4°C, followed by 10 min of enhancement with the HQ-Silver kit (Nanoprobes, Stony Brook, NY) in a dark room. After 90 min of additional fixation with 1.0% osmium tetroxide, samples were dehydrated through ethanol, acetone and QY1, and embedded in epon. Ultrathin sections of HeLa cells and motor neurons in the human spinal cord were prepared at a thickness of 70 nm and stained with uranyl acetate and lead citrate for 10 min each. The sections were observed using a JEOL 1230 transmission electron microscope (JEOL, Tokyo, Japan) and photographed with Digital Micrograph 3.3 (Gatan Inc., Warrendale, PA). Sections of 0.5 μm thickness including the spinal ventral horn were simultaneously stained with 0.3% toluidine blue to identify the resected area for observation. Images of toluidine blue staining were examined using AxioVision software (Carl Zeiss).

### Statistical analyses

Statistical significance was determined using unpaired Student’s *t*-test. *P* < 0.05 was considered statistically significant. Error bars represent the standard deviation of the mean.

## Abbreviations

lncRNA: long non-coding RNA; ncRNA: non-coding RNA; NEAT: nuclear-enriched abundant transcript; TDP-43: TAR DNA-binding protein-43; FUS/TLS: fused in sarcoma/translocated in liposarcoma; ALS: amyotrophic lateral sclerosis; ISH: *in situ* hybridization; WT: wild-type; CLIP: UV cross-linking and immunoprecipitation; IGAZ: interchromatin granule-associated zone; PSP1: paraspeckle protein-1; PSF: polypyrimidine tract binding protein-associated splicing factor; RT: reverse transcription; PCR: polymerase chain reaction; RNA-FISH: *in situ* hybridization followed by fluorescent immunohistochemistry; EM-ISH: electron microscopic analysis combined with *in situ* hybridization; ASO: antisense oligonucleotide; FITC: fluorescein isothiocyanate; DIG: digoxigenin.

## Competing interests

H. Okano is the scientific consultant of San Bio, Inc; Eisai Co Ltd; and Daiichi Sankyo Co Ltd. K. The remaining authors declare no competing financial interests. The authors declare that they have no competing interests.

## Authors’ contributions

YN, SN and SS performed all the experiments. YN, SN, TH, HJO and HO designed the experiments. TH and SN developed the RNA probes for *in situ* hybridization and TH generated rabbit polyclonal anti-PSP1 antibody. MT and SM collected to store the human spinal cords and MT provided them in appropriate conditions. SS, KK, TI provided technical assistance in immunostaining and microscopic observation. YN, SN, HJO, MT, SM, NS and HO contributed to the approvals of the Ethics Committees of institutes associated with this study. All authors contributed to the preparation of the manuscript. All authors read and approved the final manuscript.

## Supplementary Material

Additional file 1: Figure S1 Colocalization of endogenous and mutant forms of TDP-43 and FUS/TLS with NEAT1_2 lncRNA. **A.** HeLa cells were double-immunostained with polyclonal antibody against endogenous TDP-43 or FUS/TLS and monoclonal anti-DIG antibody after *in situ* hybridization using DIG-labeled NEAT1_2 probe. Endogenous TDP-43 and FUS/TLS colocalize with NEAT1_2 foci. **B**, **C**. At 48 hours after transfection with amyotrophic lateral sclerosis (ALS)-linked mutant TDP-43 and FUS/TLS with the V5 tag at the C-terminus, HeLa cells were hybridized with FITC-labeled probe against NEAT1_2 lncRNA, and immunolabeled with monoclonal anti-V5 and polyclonal anti-FITC antibodies. Aggregates formed by mutant TDP-43 and mutant FUS/TLS in the nucleus also overlap with NEAT1_2 foci. The overlapping rate among NEAT1_2 foci with aggregates formed by mutant TDP-43 or mutant FUS/TLS did not differ from that with aggregates formed by wild-type (WT) TDP-43 or FUS/TLS. Data represent mean ± s.d. The frequency was quantitatively evaluated in 50 cells for each transfection. Dotted lines represent the outline of the nucleus. **D**. Characterization of NEAT1_1 foci. Scheme of human NEAT1_1 and NEAT1_2 ncRNAs is shown at the top. Blue bars indicate probe target sites (positions 3,512–5,074 in NEAT1_2 lncRNA sequence for NEAT1_1/1_2 probe and position 14,865–15,472 for NEAT1_2 probe). At 48 hours after transfection with WT TDP-43 and WT FUS/TLS with the V5 tag at the C terminus, fixed HeLa cells were hybridized with FITC-labeled NEAT1_1/1_2 probe and double-labeled with monoclonal anti-V5 and polyclonal anti-FITC antibodies. Dotted lines represent the outline of the nucleus. Scale bars, 10 μm. Click here for file

Additional file 2: Figure S2 NEAT1_1 ncRNA is observed in the nuclei of glial cells and motor neurons in the mouse spinal cord by RNA-FISH. Left column: 8-week-old mouse spinal cord; right column: 2-y-old mouse spinal cord. Arrowheads: NEAT1_1 ncRNA in the nuclei of glial cells; arrows: faint labeling of NEAT1_1 ncRNA in the nuclei of motor neurons; dotted line: outline of the nucleus. Asterisks denote lipofuscin with autofluorescence in the cytoplasm. A long-path filter was used to distinguish Hoechst staining from autofluorescence. Scale bars, 10 μm. Click here for file

Additional file 3: Figure S3 Sense probe designed as a negative control of NEAT1_2 antisense probe in this study. The sense probe was synthesized by using the same template vector as that for the antisense probe against NEAT1_2 lncRNA and a RNA polymerase opposite to that used in synthesis of the antisense probe. Non-specific hybridization with the sense probe was not seen in human motor neurons. * Lipofuscin in the motor neuron. Scale bars, 10 μm. Click here for file

Additional file 4: Figure S4 NEAT1_2 lncRNA is often colocalized with nuclear PSF and PSP1 in ALS and control cases. RNA-FISH using DIG- or FITC-labeled NEAT1_2 probe in the nuclei of the spinal motor neurons in ALS and control cases. The right-most images show overlaps of NEAT1_2 foci and paraspeckle proteins on orthogonal sections (using ZEN software, Carl Zeiss). Dotted line: outline of the nucleus. Scale bars, 10 μm. Click here for file

Additional file 5: Figure S5 Occurrence rates of NEAT1_2 lncRNA foci in all stages of ventral motor neurons in ALS and control cases. Totally without classification, there was no difference in occurrence rates of NEAT1_2 lncRNA in all stages of motor neurons between in ALS and control cases (40.2 ± 15.5 % vs. 35.1 ± 22.6 %, respectively, *P*=0.328). Click here for file

Additional file 6: Figure S6 **A**. Electron microscopic observations combined with *in situ* hybridization (EM-ISH) in the nuclei of human spinal motor neurons using the NEAT1_1/1_2 probe (ALS and control). Using the NEAT1_1/1_2 probe, both the halo pattern of aggregation (arrows in b, e, f) and the other pattern of aggregates extending into the central portion are observed in an ALS case (a, b) and HeLa cells (e, f). Meanwhile, the halo-shaped accumulation pattern is hardly observed in a control case (c, d). HeLa cells are used as a positive control for paraspeckle formation. Scale bars, 500 nm (a, b, e, f) and 200 nm (c, d). **B**. Electron microscopic observations in a HeLa cell using diluted NEAT1_1/1_2 probe. Even when diluted NEAT1_1/1_2 probe is used, both central (a) and halo-like (b) patterns of aggregation are observed in a HeLa cell. This suggests that NEAT1_1 RNA may show the central accumulation pattern independently of the IGAZ margin. The lower panels are magnified images of the (a) and (b) regions. Scale bars, 5 μm (upper) and 200 nm (lower, a and b). **C**. EM-ISH observations in a spinal motor neuron of another case of ALS (Pt C) using the NEAT1_2 probe. Another case of ALS also shows halo-like patterns of aggregation labeled with the NEAT1_2 probe in a spinal motor neuron, similar to Pt B in Figure 6B-a and -b. Scale bars, 100 nm. Click here for file

Additional file 7: Figure S7 Prediction of FUS/TLS- and TDP-43-binding sites in NEAT1 ncRNA. Black arrowheads represent the positions (3,440–3,460; 10,108–10,127; and 18,316–18,335) where the proposed binding sites similar to the *SON* cluster are located in the NEAT1_2 genome, predicted by PAR-CLIP as preferred binding sites of FUS/TLS (ref. [27] in the text). Screening was carried out under the conditions of stem size = 6 bp and loop size = 7–9 nucleotides. Green nucleotides comprise conventional stem structures, and red nucleotides represent typical non-Watson-Crick base pairs proposed for the *SON* cluster. The yellow boxes (in positions 6,662–6,728 and 21,464–21,544) represent the binding sites of TDP-43, which have been predicted by TDP-43 iCLIP in a previous report (ref. [23] in the text). Click here for file

Additional file 8: Table S1 Oligonucleotides used in this study. Click here for file
