# Supplementary material for: The long non-coding RNA nuclear-enriched abundant transcript 1_2 induces paraspeckle formation in the motor neuron during the early phase of amyotrophic lateral sclerosis
Source: Mol Brain. 2013 Jul 8;6:31. doi: 10.1186/1756-6606-6-31 (PMC3729541; doi:10.1186/1756-6606-6-31)
Supplement: Additional file 1: Figure S1 — Colocalization of endogenous and mutant forms of TDP-43 and FUS/TLS with NEAT1_2 lncRNA. A. HeLa cells were double-immunostained with polyclonal antibody against endogenous TDP-43 or FUS/TLS and monoclonal anti-DIG antibody after in situ hybridization using DIG-labeled NEAT1_2 probe. Endogenous TDP-43 and FUS/TLS colocalize with NEAT1_2 foci. B, C. At 48 hours after transfection with amyotrophic lateral sclerosis (ALS)-linked mutant TDP-43 and FUS/TLS with the V5 tag at the C-terminus, HeLa cells were hybridized with FITC-labeled probe against NEAT1_2 lncRNA, and immunolabeled with monoclonal anti-V5 and polyclonal anti-FITC antibodies. Aggregates formed by mutant TDP-43 and mutant FUS/TLS in the nucleus also overlap with NEAT1_2 foci. The overlapping rate among NEAT1_2 foci with aggregates formed by mutant TDP-43 or mutant FUS/TLS did not differ from that with aggregates formed by wild-type (WT) TDP-43 or FUS/TLS. Data represent mean ± s.d. The frequency was quantitatively evaluated in 50 cells for each transfection. Dotted lines represent the outline of the nucleus. D. Characterization of NEAT1_1 foci. Scheme of human NEAT1_1 and NEAT1_2 ncRNAs is shown at the top. Blue bars indicate probe target sites (positions 3,512–5,074 in NEAT1_2 lncRNA sequence for NEAT1_1/1_2 probe and position 14,865–15,472 for NEAT1_2 probe). At 48 hours after transfection with WT TDP-43 and WT FUS/TLS with the V5 tag at the C terminus, fixed HeLa cells were hybridized with FITC-labeled NEAT1_1/1_2 probe and double-labeled with monoclonal anti-V5 and polyclonal anti-FITC antibodies. Dotted lines represent the outline of the nucleus. Scale bars, 10 μm. [file 1756-6606-6-31-S1.pptx]

## Slide 1
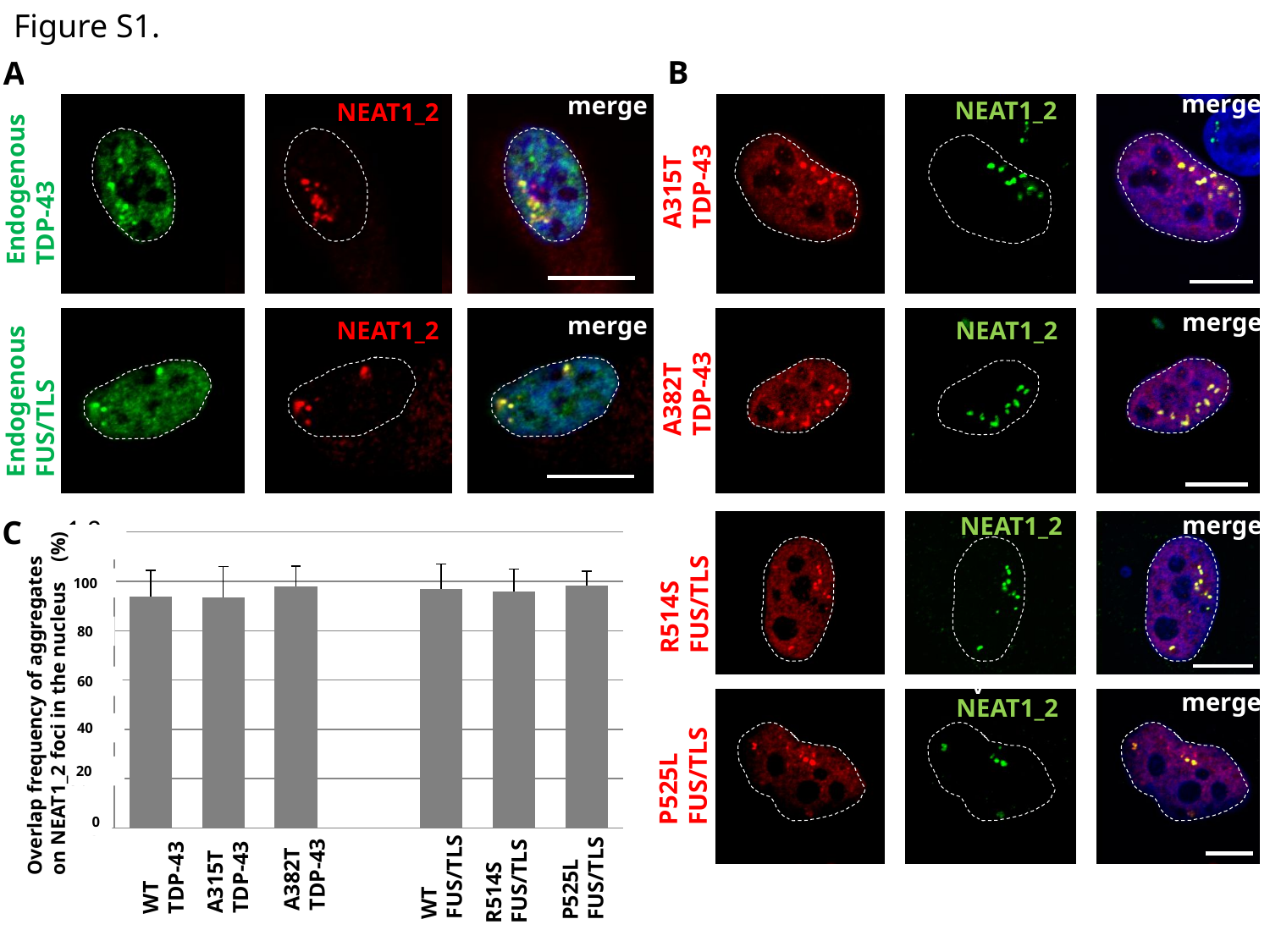

Figure S1.
B
A
merge
merge
NEAT1_2
NEAT1_2
A315T TDP-43
Endogenous
TDP-43
merge
merge
NEAT1_2
NEAT1_2
A382T TDP-43
Endogenous
FUS/TLS
merge
NEAT1_2
C
### Chart
| Category | |
|---|---|
| ① WT TDP-43 | 0.9382857142857146 |
| ④ A315T TDP-43 | 0.9337857142857148 |
| ⑤ A382T TDP-43 | 0.9796190476190481 |
| | None |
| ⑥ WT FUS | 0.9688095238095242 |
| ⑦ R514S FUS | 0.9575952380952386 |
| ⑧ P525L FUS | 0.9837777777777775 |
R514S FUS/TLS
100
80
60
Overlap frequency of aggregates
on NEAT1_2 foci in the nucleus (%)
v
merge
NEAT1_2
40
P525L FUS/TLS
20
0
A382T
TDP-43
A315T
TDP-43
WT
FUS/TLS
WT
TDP-43
P525L
FUS/TLS
R514S
FUS/TLS
v

## Slide 2
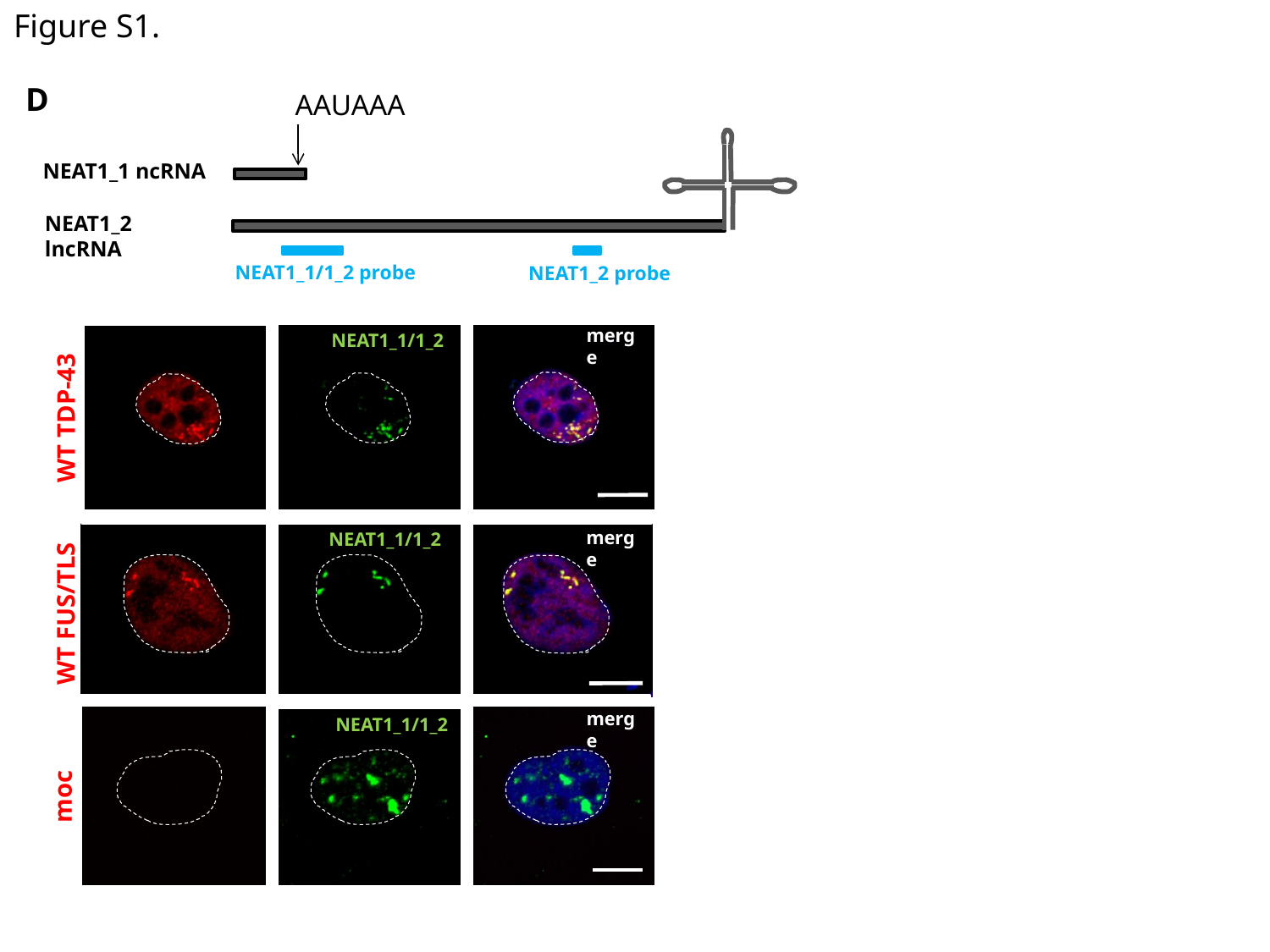

Figure S1.
D
AAUAAA
NEAT1_1 ncRNA
NEAT1_2 lncRNA
NEAT1_1/1_2 probe
NEAT1_2 probe
merge
NEAT1_1/1_2
WT TDP-43
merge
NEAT1_1/1_2
WT FUS/TLS
merge
NEAT1_1/1_2
mock
