# Supplementary material for: The long non-coding RNA nuclear-enriched abundant transcript 1_2 induces paraspeckle formation in the motor neuron during the early phase of amyotrophic lateral sclerosis
Source: Mol Brain. 2013 Jul 8;6:31. doi: 10.1186/1756-6606-6-31 (PMC3729541; doi:10.1186/1756-6606-6-31)
Supplement: Additional file 2: Figure S2 — NEAT1_1 ncRNA is observed in the nuclei of glial cells and motor neurons in the mouse spinal cord by RNA-FISH. Left column: 8-week-old mouse spinal cord; right column: 2-y-old mouse spinal cord. Arrowheads: NEAT1_1 ncRNA in the nuclei of glial cells; arrows: faint labeling of NEAT1_1 ncRNA in the nuclei of motor neurons; dotted line: outline of the nucleus. Asterisks denote lipofuscin with autofluorescence in the cytoplasm. A long-path filter was used to distinguish Hoechst staining from autofluorescence. Scale bars, 10 μm. [file 1756-6606-6-31-S2.pptx]

## Slide 1
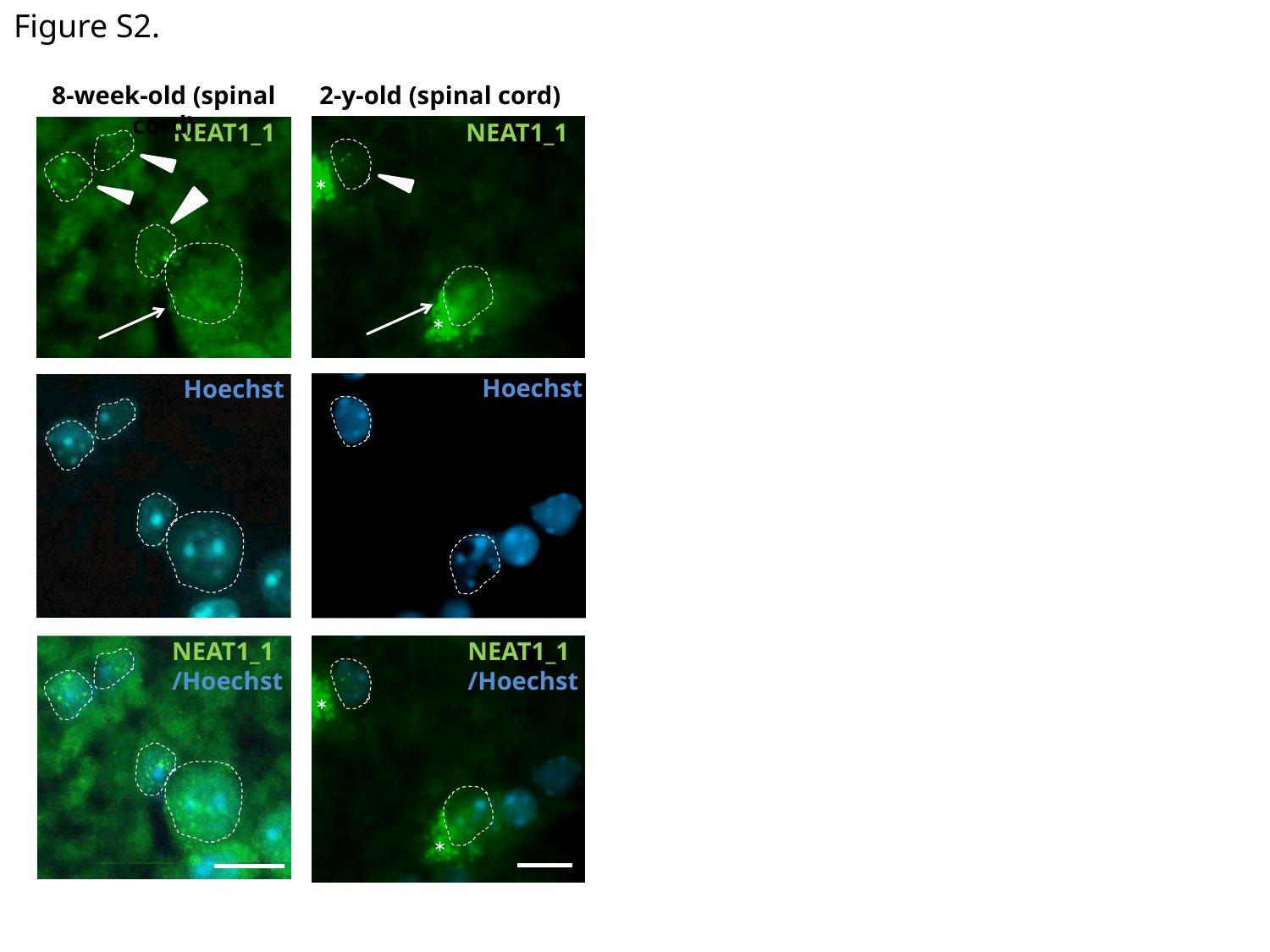

Figure S2.
8-week-old (spinal cord)
2-y-old (spinal cord)
NEAT1_1
NEAT1_1
*
*
Hoechst
Hoechst
NEAT1_1
/Hoechst
NEAT1_1
/Hoechst
*
*
