# Supplementary material for: The long non-coding RNA nuclear-enriched abundant transcript 1_2 induces paraspeckle formation in the motor neuron during the early phase of amyotrophic lateral sclerosis
Source: Mol Brain. 2013 Jul 8;6:31. doi: 10.1186/1756-6606-6-31 (PMC3729541; doi:10.1186/1756-6606-6-31)
Supplement: Additional file 3: Figure S3 — Sense probe designed as a negative control of NEAT1_2 antisense probe in this study. The sense probe was synthesized by using the same template vector as that for the antisense probe against NEAT1_2 lncRNA and a RNA polymerase opposite to that used in synthesis of the antisense probe. Non-specific hybridization with the sense probe was not seen in human motor neurons. * Lipofuscin in the motor neuron. Scale bars, 10 μm. [file 1756-6606-6-31-S3.pptx]

## Slide 1
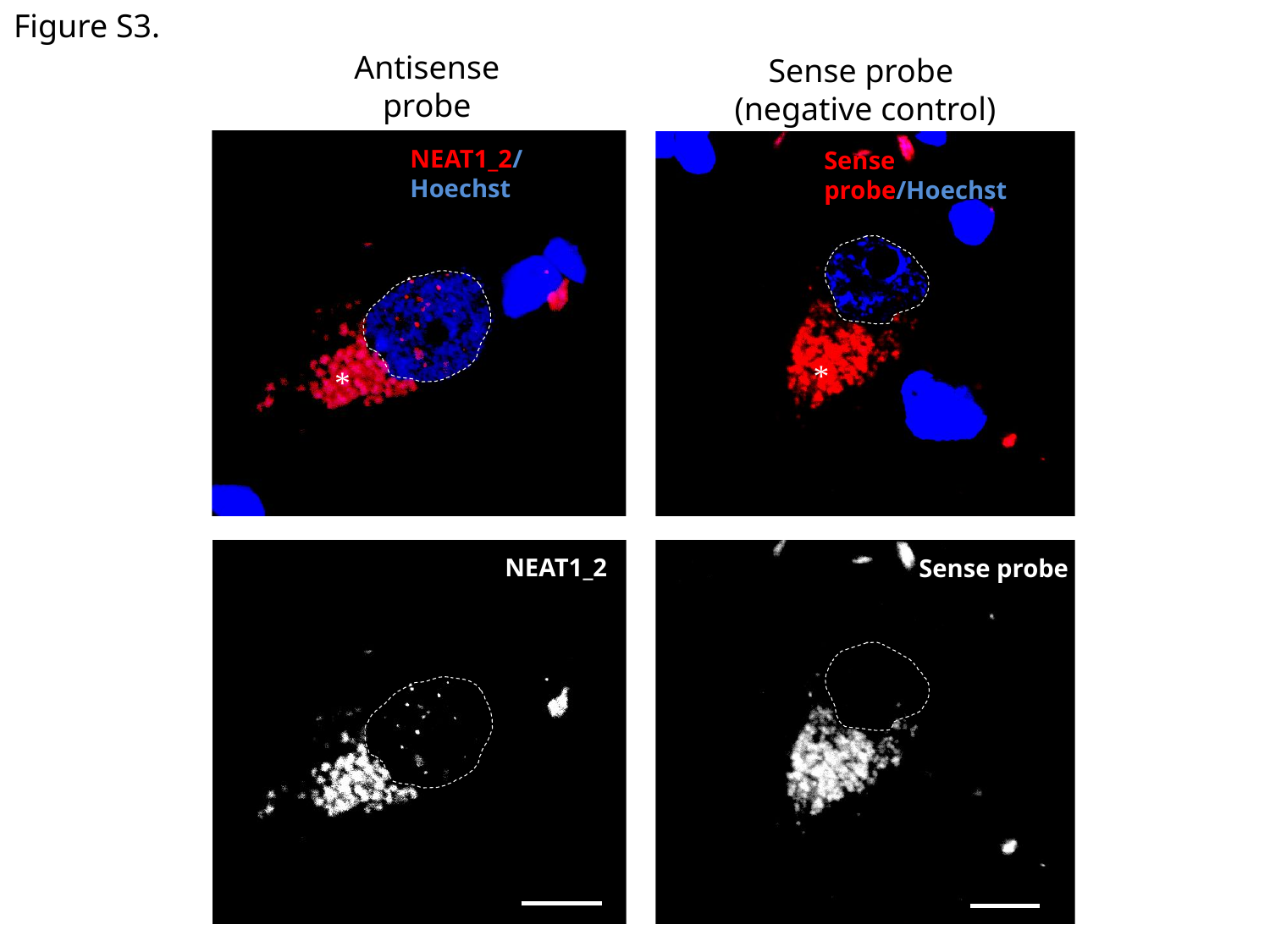

Figure S3.
Antisense probe
(NEAT1_2)
Sense probe
(negative control)
NEAT1_2/Hoechst
Sense probe/Hoechst
*
*
NEAT1_2
Sense probe
