# Supplementary material for: The long non-coding RNA nuclear-enriched abundant transcript 1_2 induces paraspeckle formation in the motor neuron during the early phase of amyotrophic lateral sclerosis
Source: Mol Brain. 2013 Jul 8;6:31. doi: 10.1186/1756-6606-6-31 (PMC3729541; doi:10.1186/1756-6606-6-31)
Supplement: Additional file 4: Figure S4 — NEAT1_2 lncRNA is often colocalized with nuclear PSF and PSP1 in ALS and control cases. RNA-FISH using DIG- or FITC-labeled NEAT1_2 probe in the nuclei of the spinal motor neurons in ALS and control cases. The right-most images show overlaps of NEAT1_2 foci and paraspeckle proteins on orthogonal sections (using ZEN software, Carl Zeiss). Dotted line: outline of the nucleus. Scale bars, 10 μm. [file 1756-6606-6-31-S4.pptx]

## Slide 1
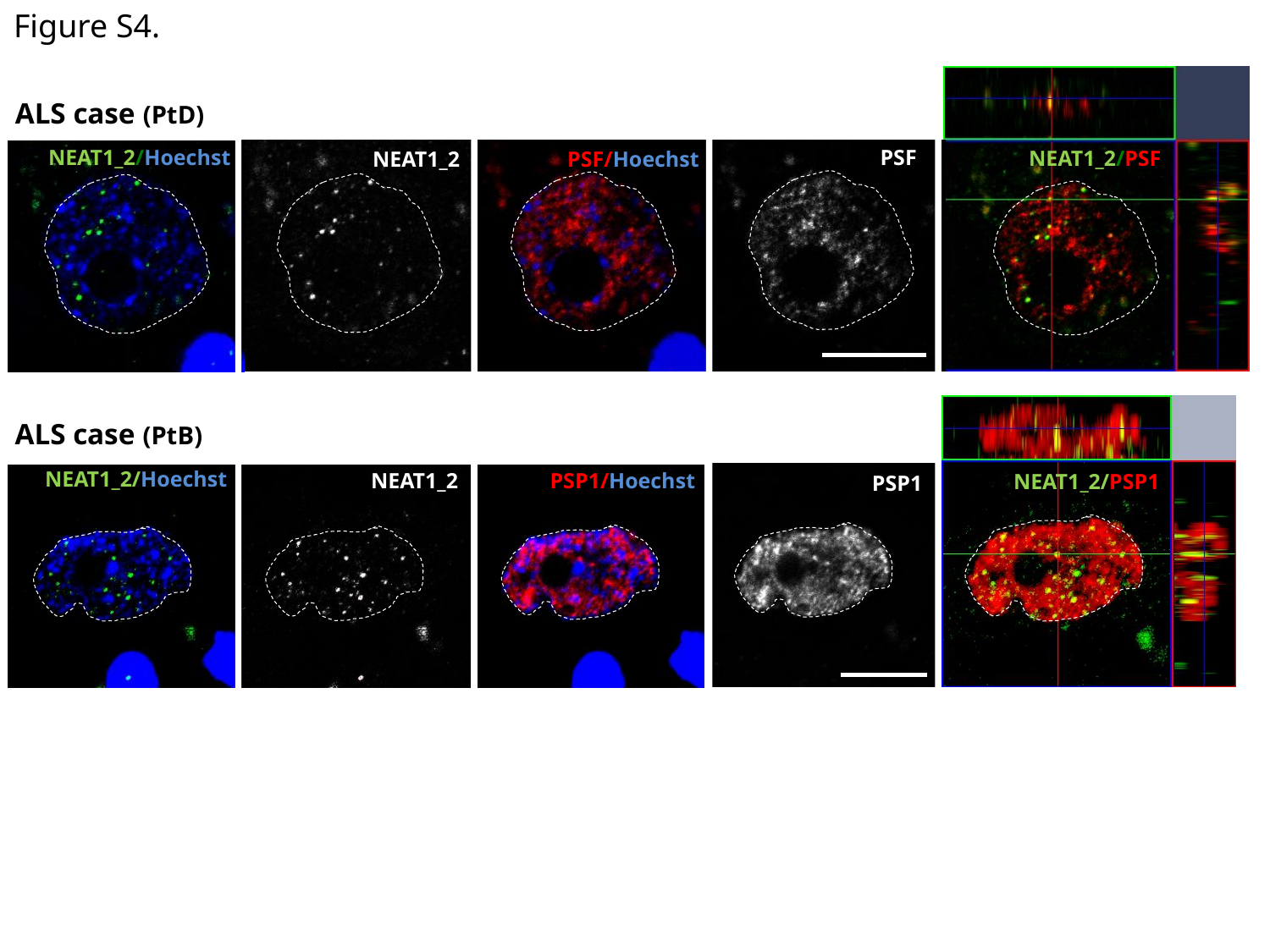

Figure S4.
ALS case (PtD)
NEAT1_2/Hoechst
PSF
NEAT1_2/PSF
NEAT1_2
PSF/Hoechst
ALS case (PtB)
NEAT1_2/Hoechst
PSP1/Hoechst
NEAT1_2
NEAT1_2/PSP1
PSP1

## Slide 2
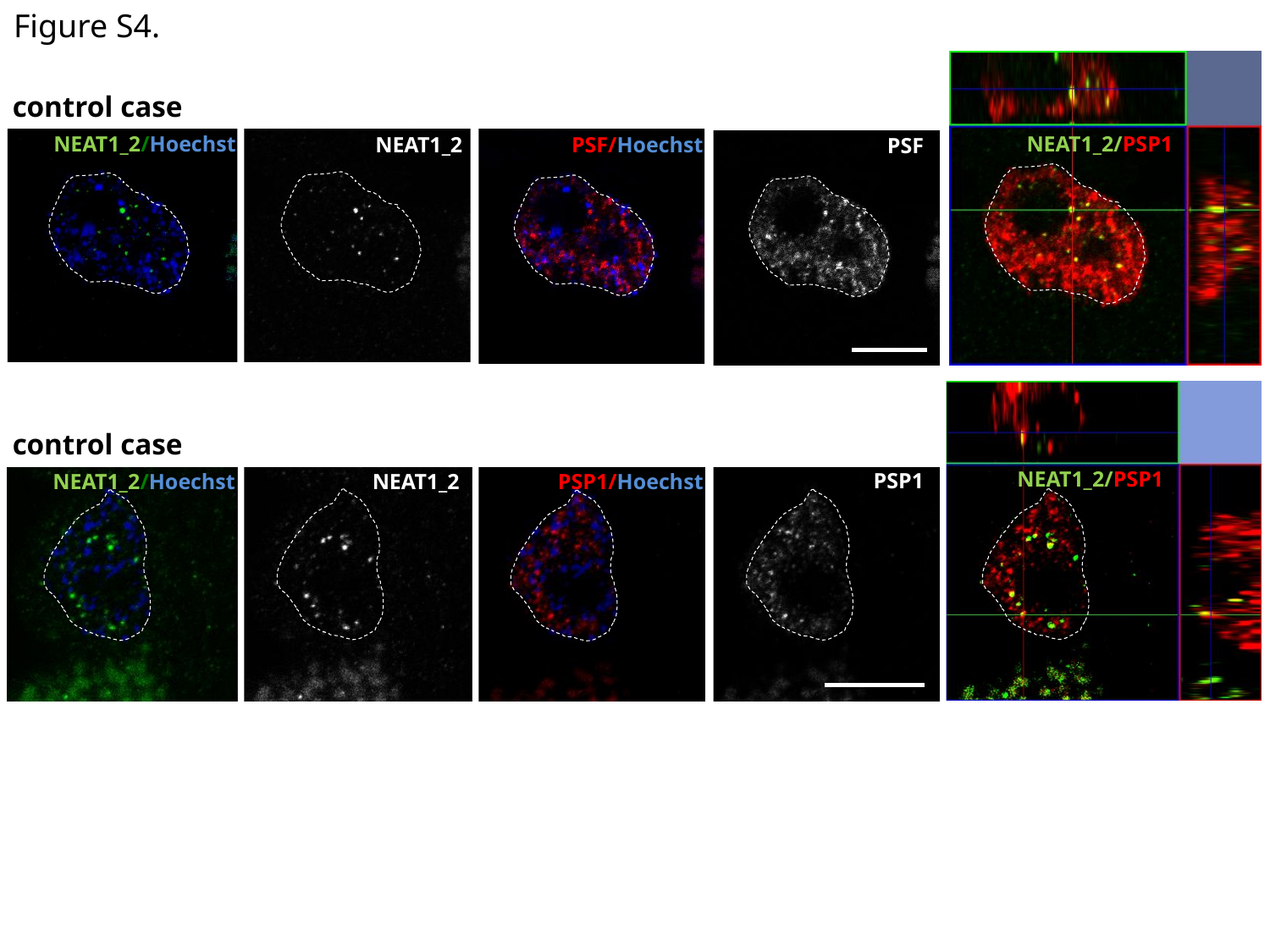

Figure S4.
control case (C4)
NEAT1_2/Hoechst
NEAT1_2/PSP1
NEAT1_2
PSF/Hoechst
PSF
control case (C2)
NEAT1_2/PSP1
PSP1
NEAT1_2
NEAT1_2/Hoechst
PSP1/Hoechst
