# Supplementary material for: The long non-coding RNA nuclear-enriched abundant transcript 1_2 induces paraspeckle formation in the motor neuron during the early phase of amyotrophic lateral sclerosis
Source: Mol Brain. 2013 Jul 8;6:31. doi: 10.1186/1756-6606-6-31 (PMC3729541; doi:10.1186/1756-6606-6-31)
Supplement: Additional file 5: Figure S5 — Occurrence rates of NEAT1_2 lncRNA foci in all stages of ventral motor neurons in ALS and control cases. Totally without classification, there was no difference in occurrence rates of NEAT1_2 lncRNA in all stages of motor neurons between in ALS and control cases (40.2 ± 15.5 % vs. 35.1 ± 22.6 %, respectively, P=0.328). [file 1756-6606-6-31-S5.pptx]

## Slide 1
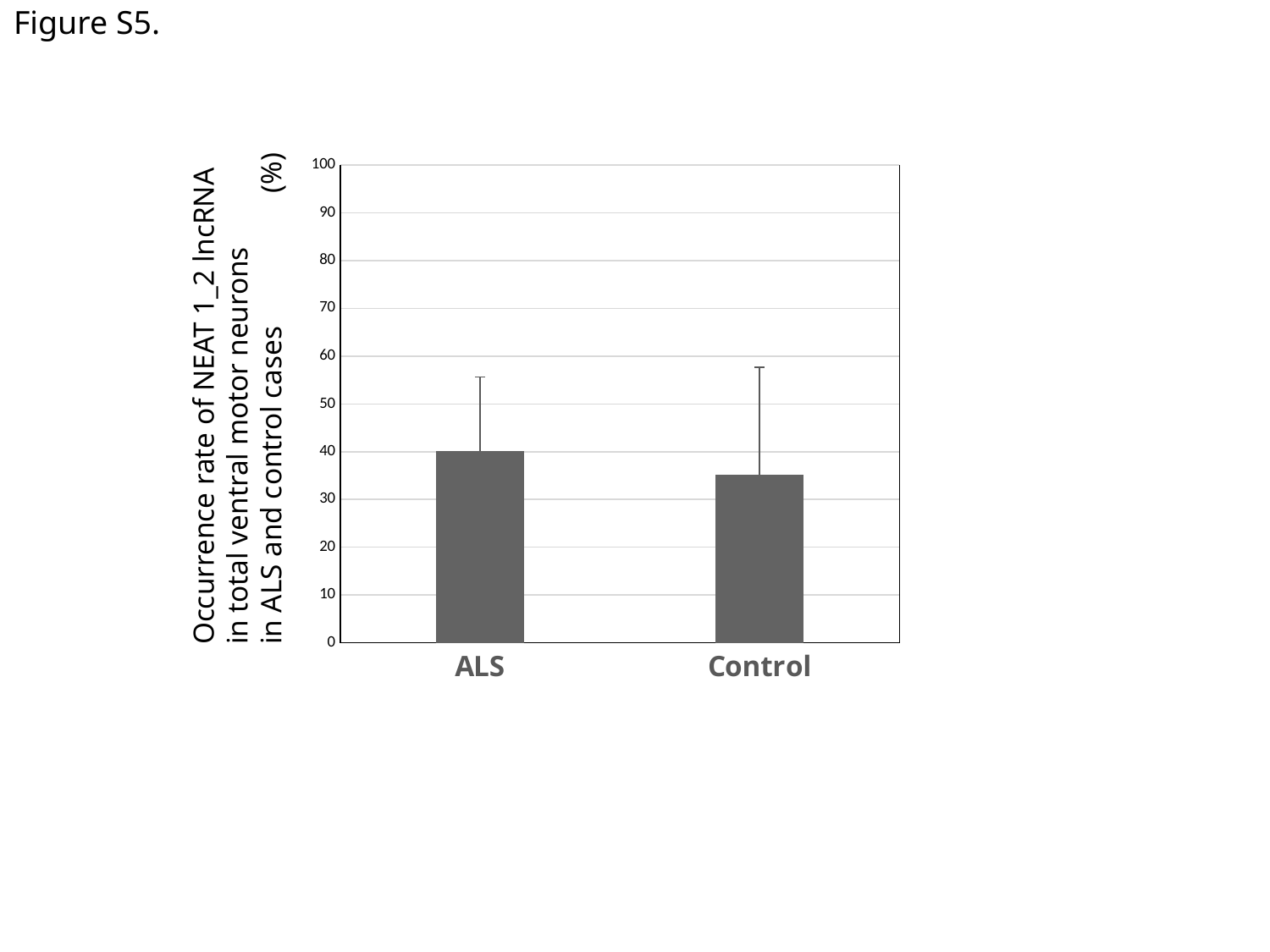

Figure S5.
### Chart
| Category | |
|---|---|
| ALS | 40.195 |
| Control | 35.075 |Occurrence rate of NEAT 1_2 lncRNA
in total ventral motor neurons
in ALS and control cases (%)
