# Supplementary figures and images for: The long non-coding RNA nuclear-enriched abundant transcript 1_2 induces paraspeckle formation in the motor neuron during the early phase of amyotrophic lateral sclerosis
Source: Mol Brain. 2013 Jul 8;6:31. doi: 10.1186/1756-6606-6-31 (PMC3729541; doi:10.1186/1756-6606-6-31)

## Slide 1
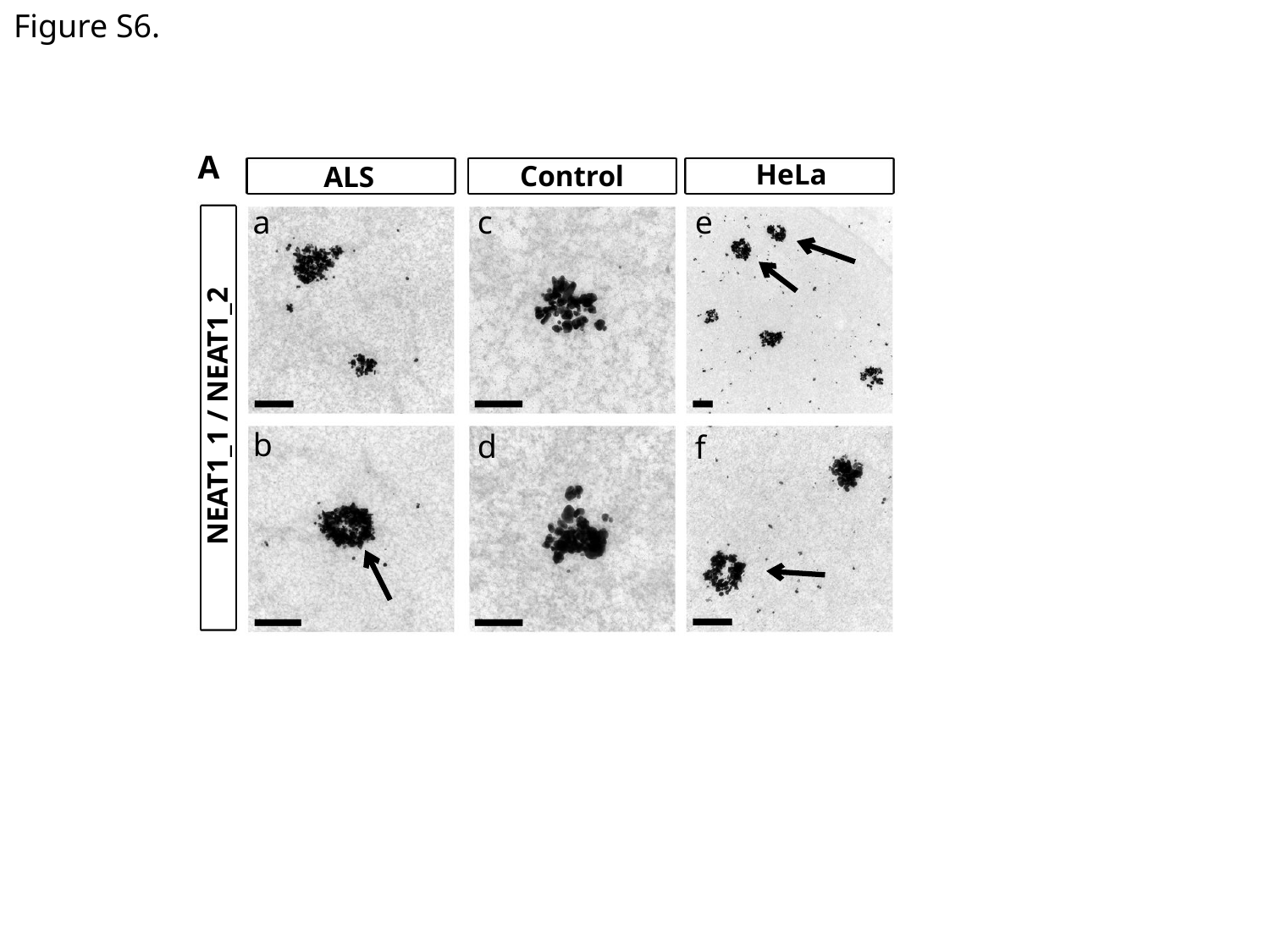

Figure S6.
A
HeLa
Control
ALS
a
c
e
NEAT1_1 / NEAT1_2
b
d
f

## Slide 2
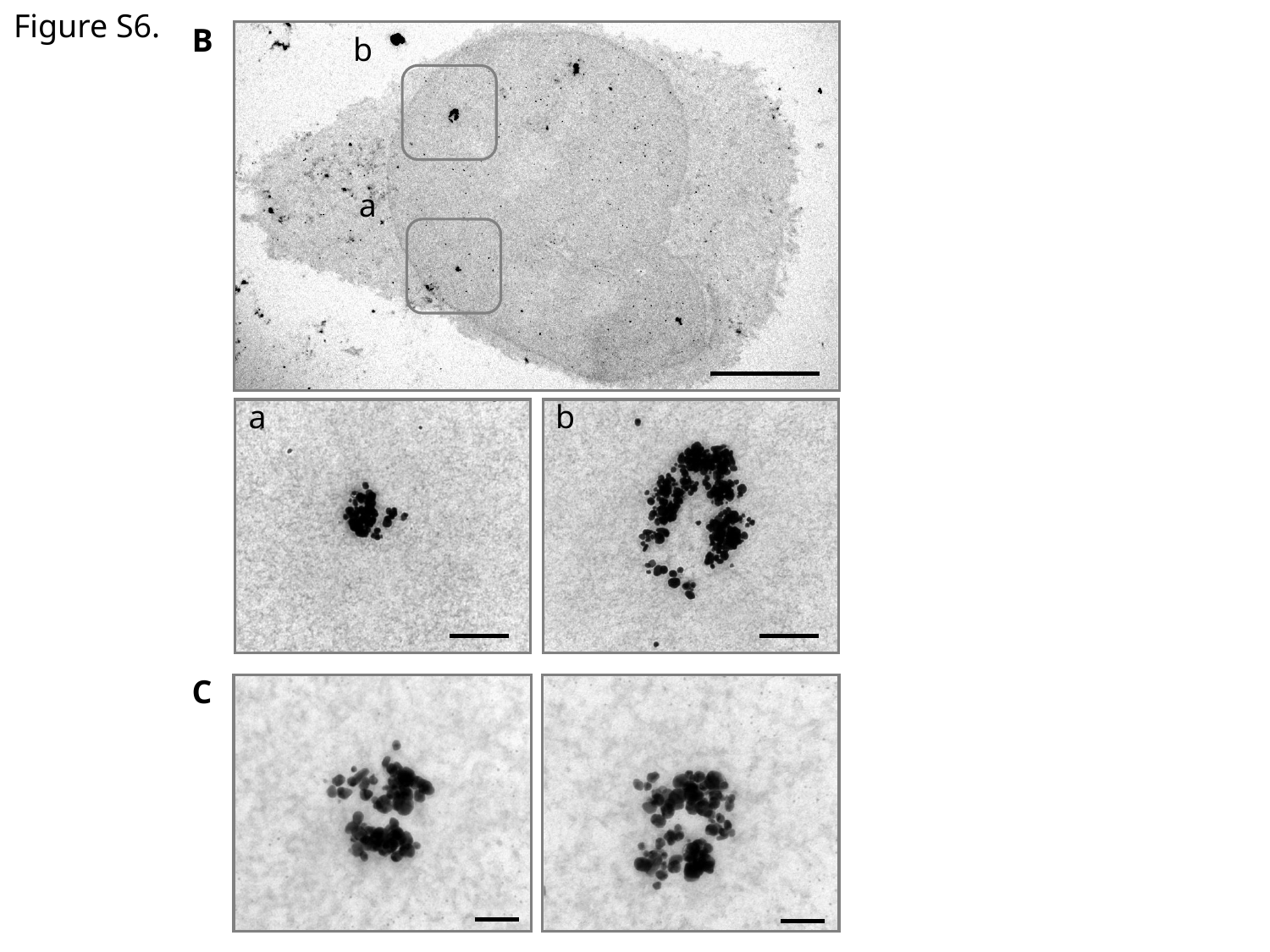

Figure S6.
B
b
a
a
b
C

Supplement: Additional file 6: Figure S6 — A. Electron microscopic observations combined with in situ hybridization (EM-ISH) in the nuclei of human spinal motor neurons using the NEAT1_1/1_2 probe (ALS and control). Using the NEAT1_1/1_2 probe, both the halo pattern of aggregation (arrows in b, e, f) and the other pattern of aggregates extending into the central portion are observed in an ALS case (a, b) and HeLa cells (e, f). Meanwhile, the halo-shaped accumulation pattern is hardly observed in a control case (c, d). HeLa cells are used as a positive control for paraspeckle formation. Scale bars, 500 nm (a, b, e, f) and 200 nm (c, d). B. Electron microscopic observations in a HeLa cell using diluted NEAT1_1/1_2 probe. Even when diluted NEAT1_1/1_2 probe is used, both central (a) and halo-like (b) patterns of aggregation are observed in a HeLa cell. This suggests that NEAT1_1 RNA may show the central accumulation pattern independently of the IGAZ margin. The lower panels are magnified images of the (a) and (b) regions. Scale bars, 5 μm (upper) and 200 nm (lower, a and b). C. EM-ISH observations in a spinal motor neuron of another case of ALS (Pt C) using the NEAT1_2 probe. Another case of ALS also shows halo-like patterns of aggregation labeled with the NEAT1_2 probe in a spinal motor neuron, similar to Pt B in Figure 6B-a and -b. Scale bars, 100 nm. [file 1756-6606-6-31-S6.pptx]
