# Supplementary material for: The long non-coding RNA nuclear-enriched abundant transcript 1_2 induces paraspeckle formation in the motor neuron during the early phase of amyotrophic lateral sclerosis
Source: Mol Brain. 2013 Jul 8;6:31. doi: 10.1186/1756-6606-6-31 (PMC3729541; doi:10.1186/1756-6606-6-31)
Supplement: Additional file 7: Figure S7 — Prediction of FUS/TLS- and TDP-43-binding sites in NEAT1 ncRNA. Black arrowheads represent the positions (3,440–3,460; 10,108–10,127; and 18,316–18,335) where the proposed binding sites similar to the SON cluster are located in the NEAT1_2 genome, predicted by PAR-CLIP as preferred binding sites of FUS/TLS (ref. [27] in the text). Screening was carried out under the conditions of stem size = 6 bp and loop size = 7–9 nucleotides. Green nucleotides comprise conventional stem structures, and red nucleotides represent typical non-Watson-Crick base pairs proposed for the SON cluster. The yellow boxes (in positions 6,662–6,728 and 21,464–21,544) represent the binding sites of TDP-43, which have been predicted by TDP-43 iCLIP in a previous report (ref. [23] in the text). [file 1756-6606-6-31-S7.pptx]

## Slide 1
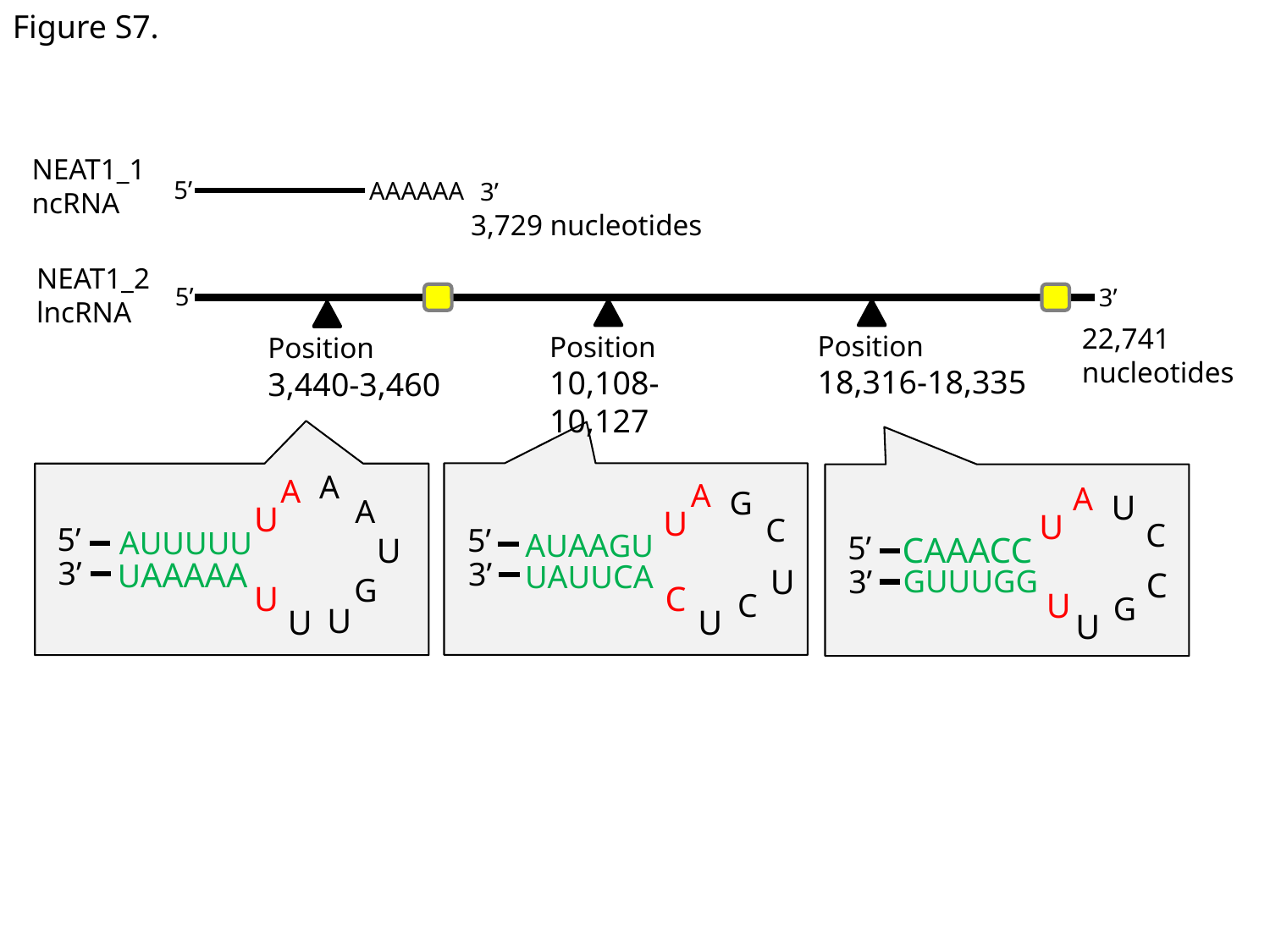

Figure S7.
NEAT1_1 ncRNA
5’
AAAAAA
3’
3,729 nucleotides
NEAT1_2
lncRNA
5’
3’
22,741 nucleotides
Position
18,316-18,335
Position
10,108-10,127
Position
3,440-3,460
A
A
A
A
G
U
A
U
U
U
C
C
5’
5’
AUUUUU
UAAAAA
AUAAGU
UAUUCA
5’
CAAACC
GUUUGG
U
3’
3’
U
3’
C
G
U
C
U
C
G
U
U
U
U
