# Supplementary material for: The long non-coding RNA nuclear-enriched abundant transcript 1_2 induces paraspeckle formation in the motor neuron during the early phase of amyotrophic lateral sclerosis
Source: Mol Brain. 2013 Jul 8;6:31. doi: 10.1186/1756-6606-6-31 (PMC3729541; doi:10.1186/1756-6606-6-31)
Supplement: Additional file 8: Table S1 — Oligonucleotides used in this study. [file 1756-6606-6-31-S8.docx]

**Table S1. Oligonucleotides used in this study**

| **Oligonucleotides** | **Sequences** |
| --- | --- |
|  |  |
| **mNEAT1_2 FW** | **TATTGGCCAATCACGAGT** |
| **mNEAT1_2 RV** | **AAAGCTGCCTGAGGCTCA** |
|  |  |
| **hNEAT1 FW** | **CAATTACTGTCGTTGGGATTTAGAGTG** |
| **hNEAT1 RV** | **CTGGAGACAGCACCATTTACATACAG** |
| **hNEAT1_2 FW** | **GTCTTTCCATCCACTCACGTCTATTT** |
| **hNEAT1_2 RV** | **CACCCTAACTCATCTTACAGACCACCAG** |
|  |  |
| **qmNEAT1 FW** | **GATCGGGACCCCAGTGACCT** |
| **qmNEAT1 RV** | **AGCTTTCCCCAACACCCACA** |
| **qmNEAT1_2 FW** | **GCTCTGGGACCTTCGTGACTCT** |
| **qmNEAT1_2 RV** | **CTGCCTTGGCTTGGAAATGTAA** |
| **qmActin FW** | **GGTGGGCCGCCCTAGGCACCA** |
| **qmActin RV** | **TTGGCCTTAGGGTTCAGGGGG** |
|  |  |
| **qhNEAT1_2 FW** | **TGTGTGTGTAAAAGAGAGAAGTTGTGG** |
| **qhNEAT1_2 RV** | **AGAGGCTCAGAGAGGACTGTAACCTG** |
|  |  |
